# Interaction of FGF9 with FGFR3‐IIIb/IIIc, a putative driver of growth and aggressive behaviour of hepatocellular carcinoma

**DOI:** 10.1111/liv.14505

**Published:** 2020-06-17

**Authors:** Jakob Paur, Maximilian Valler, Rebecca Sienel, Karin Taxauer, Klaus Holzmann, Brigitte Marian, Andreas Unterberger, Thomas Mohr, Walter Berger, Andja Gvozdenovich, Johannes Schimming, Michael Grusch, Bettina Grasl‐Kraupp

**Affiliations:** ^1^ Department of Medicine I Division: Institute of Cancer Research Comprehensive Cancer Center Vienna Medical University of Vienna Vienna Austria

**Keywords:** fibroblast growth factor 9, hepatocellular carcinoma, malignant phenotype, tumour progression

## Abstract

**Background & Aims:**

Recently, overexpression of the fibroblast growth factor receptor 3 (FGFR3) splice variants FGFR3‐IIIb and FGFR3‐IIIc was found in ~50% of hepatocellular carcinoma (HCC). Here, we aim to identify FGFR3‐IIIb/IIIc ligands, which drive the progression of HCC.

**Methods:**

FACS, MTT assay and/or growth curves served to identify the FGFR3‐IIIb/IIIc ligand being most effective to induce growth of hepatoma/hepatocarcinoma cell lines, established from human HCC. The most potent FGF was characterized regarding the expression levels in epithelial and stromal cells of liver and HCC and impact on neoangiogenesis, clonogenicity and invasive growth of hepatoma/hepatocarcinoma cells.

**Results:**

Among all FGFR3‐IIIb/IIIc ligands tested, FGF9 was the most potent growth factor for hepatoma/hepatocarcinoma cells. Replication and/or sprouting of blood/lymphendothelial cells was stimulated as well. FGF9 occurred mainly in stromal cells of unaltered liver but in epithelial cells of HCC. Every fifth HCC exhibited overexpressed FGF9 and frequent co‐upregulation of FGFR3‐IIIb/IIIc. In hepatoma/hepatocarcinoma cells FGF9 enhanced the capability for clonogenicity and disintegration of the blood and lymphatic endothelium, being most pronounced in cells overexpressing FGFR3‐IIIb or FGFR3‐IIIc, respectively. Any of the FGF9 effects in hepatoma/hepatocarcinoma cells was blocked completely by applying the FGFR1‐3‐specific tyrosine kinase inhibitor BGJ398 or siFGFR3, while siFGFR1/2/4 were mostly ineffective.

**Conclusions:**

FGF9 acts via FGFR3‐IIIb/IIIc to enhance growth and aggressiveness of HCC cells. Accordingly, blockade of the FGF9‐FGFR3‐IIIb/IIIc axis may be an efficient therapeutic option for HCC patients.

**Abbreviations**
Akt: protein kinase B
BEC: blood endothelial cells
ERK1/2: extracellular signal‐regulated kinases 1 and 2
FGF: fibroblast growth factor
FGFR: fibroblast growth factor receptor
HCC: hepatocellular carcinoma
LEC: lymphatic endothelial cells
NVP‐BGJ398 (BGJ398): selective inhibitor of FGFR1/2/3
PLCγ: phospholipase Cγ
RTK: receptor tyrosine kinase
siSCR: small‐interfering RNA targeting a scrambled DNA sequence

**Lay Summary**

Hepatocellular carcinoma (HCC) is one of the most frequent malignancies worldwide, characterized by high mortality caused by insufficient therapeutic possibilities. The present work shows that in a subgroup of HCC cases fibroblast growth factor (FGF) 9 and FGF receptor 3 variants are upregulated and that the malignant behaviour of HCC cells is driven by the interaction of FGF9 with FGFR3. Accordingly, blockade of this growth factor axis may provide a promising therapeutic target.

## 1 INTRODUCTION

Hepatocellular carcinoma (HCC) is among the three leading causes of cancer death worldwide. In industrialized countries, HCC incidences and mortalities are steadily increasing as a result of elevated prevalences of chronic hepatitis virus infections and steatohepatitis caused by ethanol abuse and/or adiposity.^1^ This malignancy is often diagnosed at an advanced stage, when local therapies are of limited efficacy. In addition, HCC are highly resistant to chemotherapy, which often leaves patients with a poor prognosis.^2^ Despite considerable toxicity, multikinase inhibitors, interfering also with FGFR1‐FGFR4, tended to improve overall survival and clinical outcomes in recent trials on advanced‐stage HCC.^3^ This is first evidence that targeting selectively FGFRs may be of great benefit, indicating urgent needs to carefully select HCC patients on the base of suitable biomarkers.

Limited information is available on the impact of the fibroblast growth factor (FGF) system on hepatocarcinogenesis.^4^ This system comprises 18 ligands with endocrine or para‐/autocrine activity and four high‐affinity cell surface tyrosine kinase receptors (FGFR1‐4).^5^, ^6^ Alternative splicing generates the receptor variants IIIb and IIIc of FGFR1‐3, which differ in the third Ig‐like domain and in their ligand specificity. According to sequence relations and gene ontology, para‐/autocrine FGFs are grouped into five FGF subfamilies. Members of these subfamilies harbour an N‐terminal signal sequence for secretion and a C‐terminal heparan sulphate‐binding site. Heparan sulphate proteoglycans, attached to the cell surface, bind the secreted FGFs, modulate their diffusion to nearby target cells and stabilize the interaction between FGFs and their receptors.^5^ Upon ligand binding, receptor monomers dimerize, followed by intracellular autophosphorylation of their kinase domains. Subsequent phosphorylation of downstream signalling components, e.g. phospholipase C gamma (PLCγ) or fibroblast growth factor receptor substrate 2 (FRS2), finally leads to activation of multiple pathways, e.g. the PI3K/AKT and the MAPK/ERK pathway, which are both involved in proliferation and migration.^5^, ^6^

Recently, we found that FGFR3‐IIIb occurs predominantly in hepatocytes and FGFR3‐IIIc in mesenchymal liver cells and that one or both splice variants are highly overexpressed in at least 50 percent of HCC cases investigated.^7^ Furthermore, aberrant expression of FGFR3 variants was causally involved in the deregulated growth control and aggressive behaviour in advanced stages of hepatocarcinogenesis.^7^ No evidence for activating mutations, translocations or amplifications at 4p16.3 could be obtained in our collective of HCC cases, indicating that deregulated transcription of FGFR3‐IIIb/IIIc combined with ligand‐induced receptor activation may be the primary mode of action.^7^ FGFR3‐IIIb binds solely FGF1 and FGF9, while FGFR3‐IIIc is open also to FGF2, FGF4, FGF8, FGF17 and FGF18, with differing affinities towards these FGFs in dependence of the test system.^4^, ^5^, ^6^, ^8^ All of the FGFR3‐IIIb/IIIc ligands, except FGF4, were found to be upregulated in subsets of the HCC cases.^7^ FGF8 subfamily members are known to induce neoangiogenesis and tumour cell survival, when cells are supplied insufficiently with oxygen and nutrients. Their impact on cell replication is low.^9^ FGF2 acts also rather as stimulator of neoangiogenesis than of growth.^4^ This raised the question which of the FGFR3‐IIIb/IIIc ligands exerts a potent growth stimulus in advanced stages of hepatocarcinogenesis.

To address this issue, FGFR3‐IIIb/IIIc ligands were tested in a panel of epithelial cell lines recently established from HCC cases.^10^ FGF9 was found to be the most potent growth stimulator of the hepatoma/hepatocarcinoma cells and to enhance also the invasive/migratory phenotype of the cells. These effects appear to be mediated via FGFR3‐IIIb/IIIc. FGF9 is expressed mainly in the stroma of unaltered liver and overexpressed in the epithelial cells of every fifth HCC, indicating a switch from a paracrine to autocrine mode of action. Most of the FGF9‐positive tumours exhibited also elevated FGFR3‐IIIb or FGFR3‐IIIc. To conclude, deregulations of the FGF9‐FGFR3‐IIIb/IIIc axis appear to drive growth and progression of hepatic malignancy.

## 2 MATERIALS AND METHODS

### 2.1 Human liver samples

Patients were subjected to surgical resection of HCC, metastasis or other intrahepatic alterations (Table S1A/B). Written informed consent was obtained from each patient. No donor organs were used from executed prisoners or other institutionalized persons. The study protocol conformed to the ethical guidelines of the 1975 Declaration of Helsinki, as reflected by the approval of the “Ethic Committee of the Medical University of Vienna” (approval number 479/2002).

### 2.2 Cell lines and treatment

HepG2 and Hep3B cells were obtained from ATCC (Rockville, MD). The hepatocarcinoma cell lines (HCC‐1.1, HCC‐1.2, HCC‐2 and HCC‐3) and telomerase‐immortalized human lymphendothelial (LEC) and blood endothelial cell lines (BEC) were recently established and characterized.^10^, ^11^ At regular intervals aliquots were taken from cryopreserved cell line stocks, being authenticated by STR profiling. BGJ398 was dissolved in DMSO and applied at 500 nM (Hep3B, HCC‐1.2 and HCC‐3) or 1 µM (HepG2).^12^

### 2.3 Immunohistochemistry

Sections, obtained from formalin‐fixed liver tissue, were stained for FGF9, cd34 and α‐smooth muscle actin, as described in detail before.^7^

### 2.4 Reverse transcription quantitative PCR (RT‐qPCR)

mRNA was extracted from cells, processed and subjected to the ABI‐Prism‐PCR standard protocol (ABI‐Prism‐7500‐Sequence‐Detection‐System) using ABI‐Prism‐7500‐SDS software (Applied Biosystems), as described.^7^, ^9^ For further details see Table S2.

### 2.5 Immunoblotting

Protein purification, separation and detection followed published protocols.^7^, ^9^ The blots were probed with specific antibodies, as specified in Table S2.

### 2.6 Clonogenicity assay

Cells were plated at densities of 100 (Hep3B), 170 (HCC‐1.2, HepG2) or 250 (HCC‐3) cells/cm^2^. When clones appeared in controls, cells were fixed in acetone/methanol (v/v 1:1), stained with 0.01% of crystal violet and quantified by ‘LUCIA G image analyser’ (Nikon). The cloning efficiency was determined as the percentage of the cells seeded that had formed a clone.

### 2.7 Analyses of viability, apoptosis and cell cycle

Numbers of viable cells were determined by the 3‐(4,5‐dimethylthiazol‐2‐yl)‐2,5‐diphenyltetrazolium bromide (MTT) assay (EZ4U; Biomedica, Vienna, Austria). To quantify apoptosis, cells were incubated in 0.5 mL medium containing 0.6 μg/mL propidium iodide (Sigma‐Aldrich) and analysed by FACSCalibur (Becton‐Dickinson). For cell cycle analysis by FACSCalibur, cells were fixed in 70% ethanol, RNA was digested with RNAse A (10 mg/mL; Qiagen, Hilden, FRG) and DNA was stained with propidium iodide (2.5 μg/mL). DNA synthesis was assayed by ^3^H‐thymidine incorporation and scintillation counting, as described.^9^, ^10^

### 2.8 Small‐interfering RNA (siRNA) knockdown

siRNAs were transfected at 10 nmol (HCC‐1.2, HCC‐2 and HCC‐3) or 20 nmol (Hep3B and HepG2) using siLentFect (BioRad) according to the manufacturer's instructions.

### 2.9 CCID (circular chemorepellent‐induced defects) assay

Three × 10^3^ cells were incubated in 150 µl medium containing 20% methylcellulose (M‐0512, Sigma‐Aldrich) in round bottom microtitre plates to allow spheroid formation within 48 hours. Spheroids were washed in EGM2 medium (Lonza, Walkersville, MD), transferred to confluent BEC or LEC monolayers expressing mCherry and kept in EGM2 medium; 3 hours (Hep3B), 4 hours (HCC‐1.2) or 5.5 hours (HepG2) later, the spheroid and the gap area in the BEC/LEC monolayer underneath were photographed. For each condition, the 2D size of > 10 spheroids and appendant gaps were determined by ImageJ software (National Institutes of Health). Further detail see.^11^

### 2.10 Tube formation assay

BEC and LEC were seeded onto growth factor‐reduced Matrigel (Becton Dickinson). Fourteen hours after addition of FGFs, the extent of tube formation was quantified by determining the length of tubes and the extent of branching (number of nodes) using ImageJ software, as described.^9^

### 2.11 Spheroid sprouting assay

The assay followed principally previous descriptions.^13^ Two ×10^5^ BEC or LEC were suspended in 2.2 ml 70% (V/V) high viscosity methylcellulose and 8.8 ml (V/V) EGM2 medium and incubated as hanging drop of 25 µl; 24 hours later spheroids were harvested, resuspended in 2 ml of 70% high viscosity methylcellulose + 30% EBM2‐medium (Lonza) and mixed with 3.2 mL rat collagen + 400 µL 10 × M199‐medium (Sigma‐Aldrich), neutralized with 0.2 N sodium hydroxide. The spheroid/collagen mixture was placed onto 24‐well plates for 60 minutes and was covered with EBM2‐medium + 0.05% FCS with or without FGF9 or VEGF. Twenty‐four hours later cells were fixed in 10% (m/V) formaldehyde for the analysis of the sprout length by ImageJ software.

## 3 RESULTS

### 3.1 FGF9, a potent growth stimulator of hepatoma/hepatocarcinoma cell lines

**FIGURE 1:**
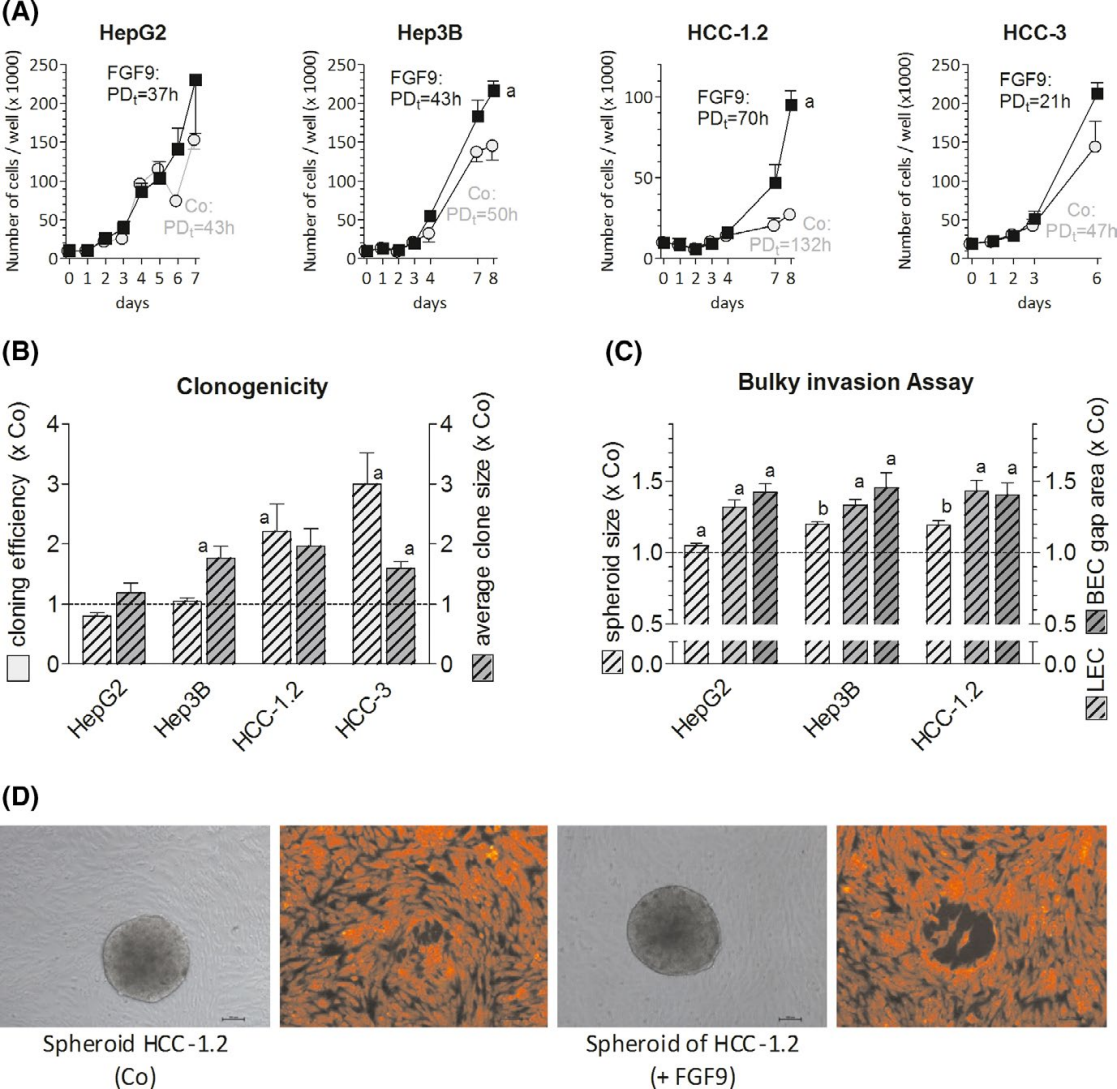
FGF9 enhances growth, cloning efficiency and the invasive phenotype of hepatoma/hepatocarcinoma cells. (A), Twenty‐four h after seeding cells were treated with 10 ng of FGF9/ml medium. Cells were counted at regular intervals; the population doubling time (PD_t_) was calculated using the first and last count. (B), Cell lines were plated at low densities and were treated with 10 ng of FGF9/ml medium. Clone numbers and size were determined after 10 d (HepG2) or 2 wk (Hep3B, HCC‐1.2, HCC‐3). (C,D), Spheroids were formed (for details see Methods) and were placed on confluent LEC and BEC monolayers for~4 h. Pictures of spheroids and gaps in monolayers underneath were taken and sizes were measured by ImageJ software. (D), Representative spheroids (in gray) of HCC‐1.2 cells and corresponding gaps formed beneath in LEC (red fluorescence). (A‐C), All data are given as means ± SEM of ≥3 independent experiments. Statistics in (A) by paired *t*‐test for FGF9 vs untreated control (Co) on the last day: a, *P* < .05; statistics in (B) and (C) by One Sample *t*‐test for FGF9 treatment vs Co: a, *P* < .05; b, *P* < .01

We chose a panel of hepatoma/hepatocarcinoma cells, reflecting the variable upregulation of FGFR3‐variants in HCC, i.e. Hep3B and HCC‐2 express rather isoform IIIb, HCC‐1.2 and HCC‐3 cells predominantly FGFR3‐IIIc, and HCC‐1.1 and HepG2, both variants at more or less similar extent (Table S3). The relative growth inducing potency of FGFR3‐IIIb/IIIc ligands was strongest for FGF9 followed by FGF1, FGF18, FGF4, FGF17, FGF8 and finally FGF2 (Figure S2). Accordingly, FGF9 was found to reduce the population doubling time in most cell lines tested (Figure 1A). This was partly caused by shifts towards the S/G2‐M phase of the cell cycle and/or somewhat lowered apoptotic activities, contributing to net gains of hepatoma/hepatocarcinoma cells (Table S4).

### 3.2 FGF9 enhances the aggressive phenotype of hepatoma/hepatocarcinoma cells

FGF9 enhanced significantly the clone‐forming capacity of all hepatoma/hepatocarcinoma lines except for HepG2 cells (Figure 1B). In a three‐dimensional assay for bulky invasion, HepG2, Hep3B and HCC‐1.2 cells formed considerably larger spheroids when treated with FGF9 than in controls, probably as a consequence of elevated cell proliferation. Furthermore, FGF9 raised the cells´ capacity to induce gaps in monolayers of blood or lymphatic endothelium (Figure 1C/D). In addition, FGF9‐treated Hep3B and HCC‐1.2 cells migrated faster in a wound‐healing assay, but not HepG2 cells (Figure S3). The reasons for the deviant reactivity of HepG2 cells towards FGF9 are presently unclear since this line showed a similar FGF9‐induced phosphorylation pattern of signal transduction components when compared to the other lines (see below).

Overall, these data show that FGF9 acts as a potent growth factor for hepatoma/hepatocarcinoma cells and appears to enhance the cells´ ability for clone formation, migration and disintegration of blood/lymphatic endothelia.

### 3.3 FGF9 supports growth of tumour stroma cells and neoangiogenesis

**FIGURE 2:**
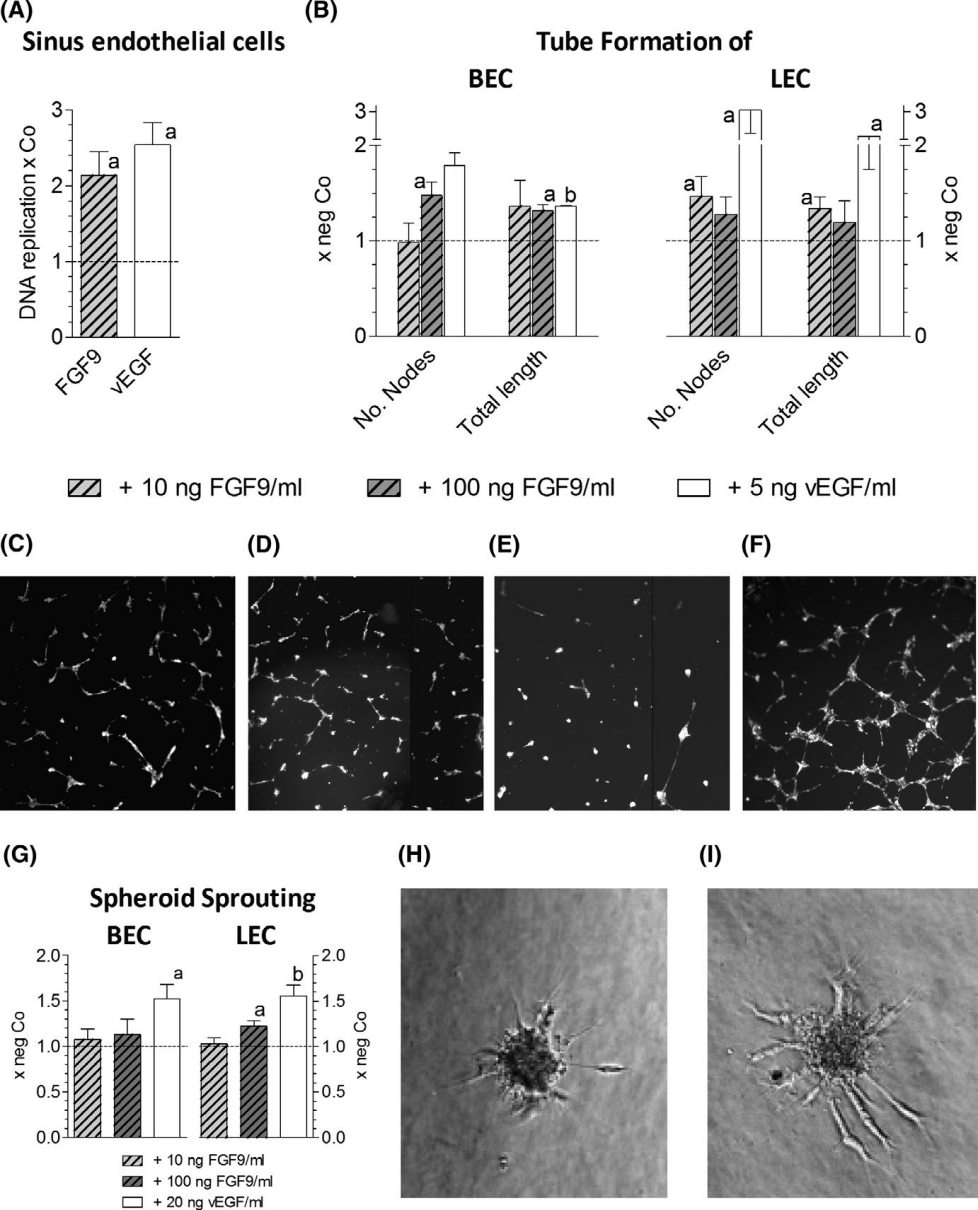
FGF9 stimulates neoangiogenesis. (A), Freshly isolated rat liver sinus endothelial cells were kept under standard conditions, as described,^14^ and were treated with 10 ng of FGF9/ml or 5 ng vEGF/ml medium; 24 h later DNA replication was determined by ^3^H‐thymidine incorporation and scintillation counting. (B‐F), Tube formation of BEC and LEC was induced by treatment with FGF9 for 14 h, as described in methods. BEC: untreated (C) or treated with 10ng FGF9/ml (D); LEC: untreated (E) or treated with 100ng FGF9/ml (F). (G‐I), Spheroid sprouting assay was performed as described in methods. Sprouting BEC spheroid, being untreated (H) or treated with 100 ng of FGF9/ml medium (I). Data in (A), (B) and (G) give means ± SEM of ≥3 three independent experiments. Statistics by One Sample *t*‐test: a, *P* < .05; b, *P* < .01

In response to pro‐angiogenic stimuli tumour vessels form capillaries via stalk cells, which proliferate and adjust behind tip cells to extend the sprouts and to form a network of tubes. The impact of FGF9 on neoangiogenesis was studied in freshly isolated sinus endothelial cells, BEC, LEC and VEGF‐producing tumour‐associated myofibroblast cell lines, recently established from HCC cases (Figure S4A).^10^, ^14^ FGF9 elevated DNA replication of sinus endothelial cells and partly also of myofibroblasts (Figure 2A, S4B). In addition, FGF9 enhanced the capacity of LEC cells to form tubes and nodes (bifurcations) at 10 ng/ml medium and of BEC at 100 ng/ml medium (Figure 2B‐F). Also the sprout formation of LEC was increased by FGF9 (Figure 2G‐I). This suggests that in HCC FGF9 may support the formation of new blood and lymphatic capillaries directly as well as indirectly via multiplication of VEGF‐secreting myofibroblasts.

### 3.4 Co‐occurrence of FGF9 and FGFR3‐IIIb/IIIc overexpression in HCC

**FIGURE 3:**
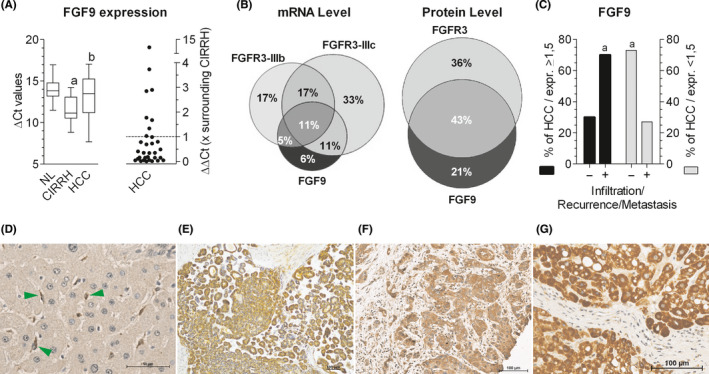
Frequent co‐occurrence of upregulated FGF9 and FGFR3 in hepatocellular carcinoma: possible implications for infiltrative growth. (A, left panel), FGF9 mRNA levels were determined by RT‐qPCR for normal (NL) or cirrhotic livers (CIRRH) and hepatocellular carcinoma (HCC). (A, right panel), mRNA levels of individual HCC samples were expressed as ‐fold surrounding (non‐tumour) liver tissue, obtained from the same patient: values > 1 indicate a higher expression level in HCC than in surrounding tissue. (B), Venn diagrams show HCC cases with upregulation of FGF9, FGFR3‐IIIb and/or FGFR3‐IIIc at the mRNA level (set 100%; left panel) and of FGF9 and/or FGFR3 at the protein level (set 100%; right panel). (C), Black bars stand for all HCC cases with overexpression of FGF9 protein (set 100%), grey bars give all HCC cases without overexpression of FGF9 protein (set 100%). Within these two categories, cases were grouped according to: ‐, no local invasion and/or metastasis at the time point of resection and/or no recurrence within 82 ± 20 months of follow‐up; +, local invasion and/or metastasis at the time point of resection and/or recurrence within 9 ± 17 months of follow‐up. Further detail see Table S1. FGF9 immunostains of paraffin‐embedded tissue sections: (D), Unaltered liver with FGF9‐positive mesenchymal cells (green arrows); (E‐G), Details of HCC tissue of three different cases. (A), Boxplot: whiskers at minimum and maximum, box at 25th percentile and 75th percentile with a line at 50th percentile. Statistics by unpaired *t*‐test for NL vs CIRRH: a, *P* < .01; for CIRRH vs HCC: b, *P* < .05. Statistics in (C) by Chi‐square test: a, *P* < .05

Compared to surrounding cirrhotic liver tissues, we found elevated FGF9 transcripts in 6 of 32 HCC cases (19%) (Figure 3A). Highly similar data were derived from a large collective of 404 HCC cases, obtained from Genomic Data Commons Data Portal of the National Cancer Institute showing that approximately every third HCC overexpresses FGF9 (Figure S5).

In our collective the findings on the transcript level were confirmed at the protein level by positive immunostaining of 18 HCC (33%) among 53 cases investigated (Figure 3E‐G, S1C‐H, S6E‐F; Table S1A). In detail, surrounding liver tissue revealed faint immunoreaction of hepatocytes and more pronounced staining of cells lining the sinusoids. Immunostained serial sections showed FGF9‐positive cells being positive also for α‐smooth muscle actin, a marker for activated stellate cells (Figure S6A‐D). This finding was confirmed by RT‐qPCR demonstrating the highest FGF9 expression in stellate cells (Figure S7A‐B). In contrast, all of the FGF9 overexpressing HCC cases revealed positive tumour parenchyma (Figure 3E‐G, S1C‐H and S6E‐F) and only occasionally FGF9‐positive tumour stroma cells. This provides evidence for a mesenchymal‐to‐epithelial switch of FGF9 expression during hepatocarcinogenesis.

Most of the FGF9‐positive HCC exhibited also elevated levels of FGFR3‐IIIb or FGFR3‐IIIc mRNA causing that 82% of the tumours with overexpression of FGF9 also showed upregulated FGFR3‐IIIb and/or FGFR3‐IIIc (Figure 3B, Table S1). There was also co‐occurrence of elevated FGF9 and FGFR3 protein in 67% of all FGF9‐positive HCC.

The analysis of clinical data showed no association between the extent of FGF9 overexpression with cause of disease, size or grade of HCC (TableS1). However, local infiltration, metastatic tumour spread at the time of tumour resection and/or within on average 9 months of follow‐up was associated with enhanced expression of FGF9 (Figure3C). This indicates that upregulated FGF9 might be associated with tumour infiltration and/or recurrence.

### 3.5 FGF9 may act in HCC and hepatoma/hepatocarcinoma cells mainly via FGFR3‐IIIb and/or FGFR3‐IIIc

In dependence of the test system FGF9 may exert effects mostly via FGFR3‐IIIb/IIIc and FGFR2‐IIIc, to some extent via FGFR1‐IIIc and marginally via FGFR4 and FGFR1‐IIIb.^8^ Similar to hepatocytes, hepatoma/hepatocarcinoma cell lines express all FGFRs (Table S5). In these lines FGFR1‐4 were knocked down by siRNA to test whether these receptors may be involved in the FGF9 action. By this approach, the IIIb/IIIc splice variants were affected to a similar extent (not shown) and with high receptor specificity (Figure S8). Silencing of FGFR1 and FGFR2 did not interfere significantly with the FGF9‐specific phosphorylation pattern in the signal transduction cascade (Figures S9‐S11A) or the FGF9‐enhanced formation and growth of clones (Figure S11B). Also FGFR4 knockdown did not impact significantly on replication, apoptosis or clone formation of the cells (Figure S12). This was first and indirect evidence that in the hepatoma/hepatocarcinoma cells FGF9 acts largely via FGFR3‐IIIb/IIIc.

**FIGURE 4:**
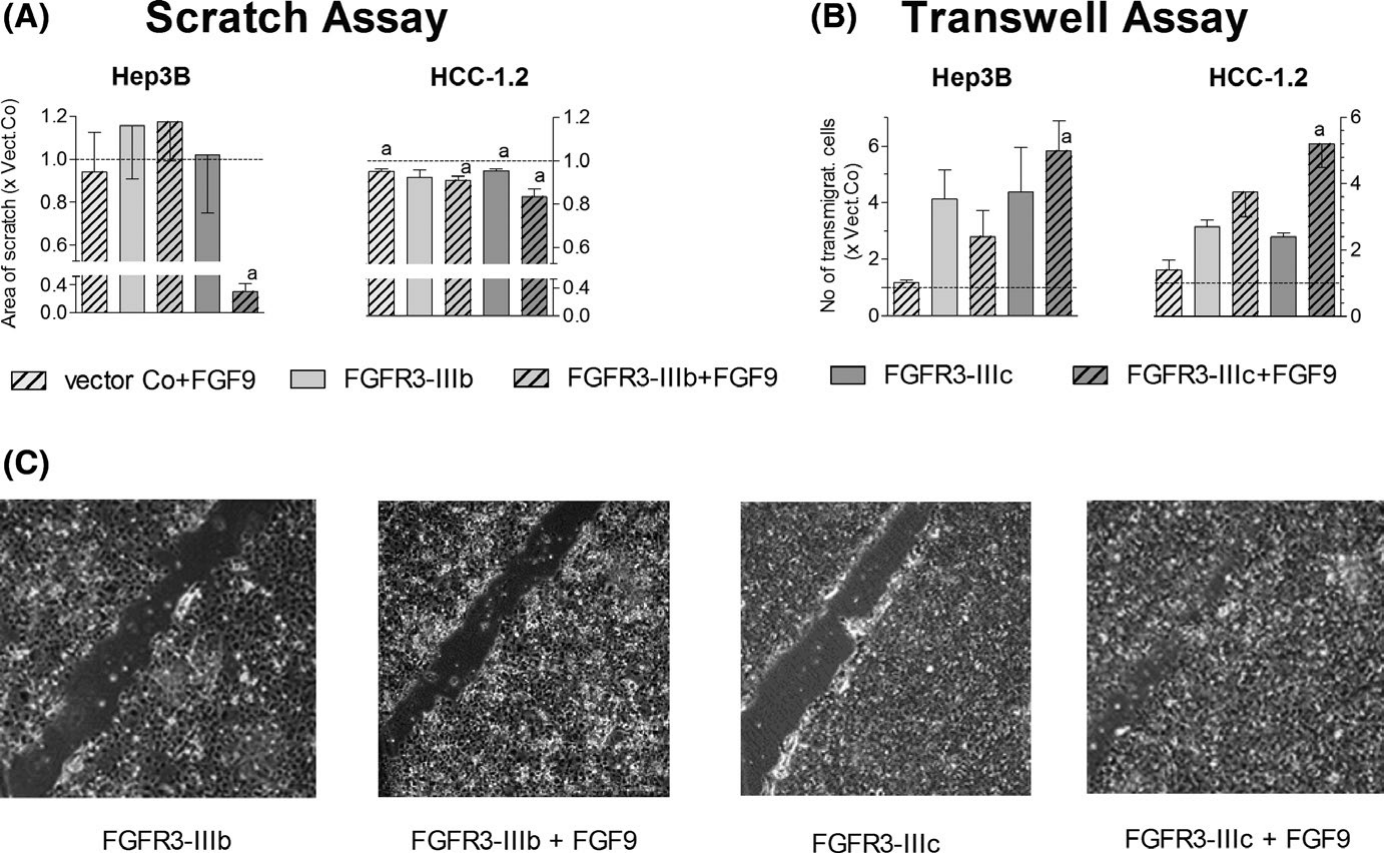
FGF9 enhances the migration of hepatoma/hepatocarcinoma cells over‐expressing FGFR3‐IIIc. (A), Cells, over‐expressing either the control vector or FGFR3‐IIIb/IIIc, were grown to confluence and switched to serum‐free medium. Monolayers were scratched by 200μl pipette tips, followed by rinsing and treatment with 10 ng FGF9/ml medium. After 48 h the total area of scratches was measured by ImageJ software. (B), 2 x 10^4^ cells were seeded onto cell culture migration inserts. The cells, which had migrated through the insert within 48 h, were fixed, stained with crystal violet and counted. (C), Pictures of representative scratch assays with Hep3B cells are given. (A‐B), Data are expressed as mean ± SEM of 2‐4 independent experiments. Statistics by One sample *t*‐test: a, *P* < .05

Considering that >80% of FGF9‐positive HCC show enhanced expression of FGFR3‐IIIb and/or FGFR3‐IIIc, FGF9 may exert strong effects in cells with upregulated FGFR3 variants. Cell lines with stable overexpression of either FGFR3‐IIIb or FGFR3‐IIIc had been generated (Figure S13).^7^ As shown recently, FGFR3‐IIIb confers the ability for enhanced proliferation, and FGFR3‐IIIc supports the disintegration of the blood/lymphatic endothelium and cell migration.^7^ In response to FGF9, FGFR3‐IIIb overexpressing hepatoma/hepatocarcinoma cell lines formed even larger clones than the vector controls (Figure S14), while FGF9‐treated cells with overexpressed FGFR3‐IIIc showed an elevated migratory phenotype, as indicated by the wound‐healing and transwell assay (Figure 4).

To conclude, these data together provide evidence that the FGF9 effects on hepatoma/hepatocarcinoma cells may be mediated mainly by FGFR3‐IIIb/IIIc.

### 3.6 Blockade of the FGF9‐FGFR3 axis reduces the aggressive behaviour of hepatoma/hepatocarcinoma cells

To block the interaction of FGF9 with FGFR3, two different approaches were chosen, i.e. siRNA‐mediated down‐modulation of the receptor and inhibition of the tyrosine kinase activity. Owing to lack of FGFR3‐specific tyrosine kinase inhibitors, BGJ398 was applied, affecting wild‐type FGFR1‐3 and a common FGFR3 mutant (S249C).^12^

**FIGURE 5:**
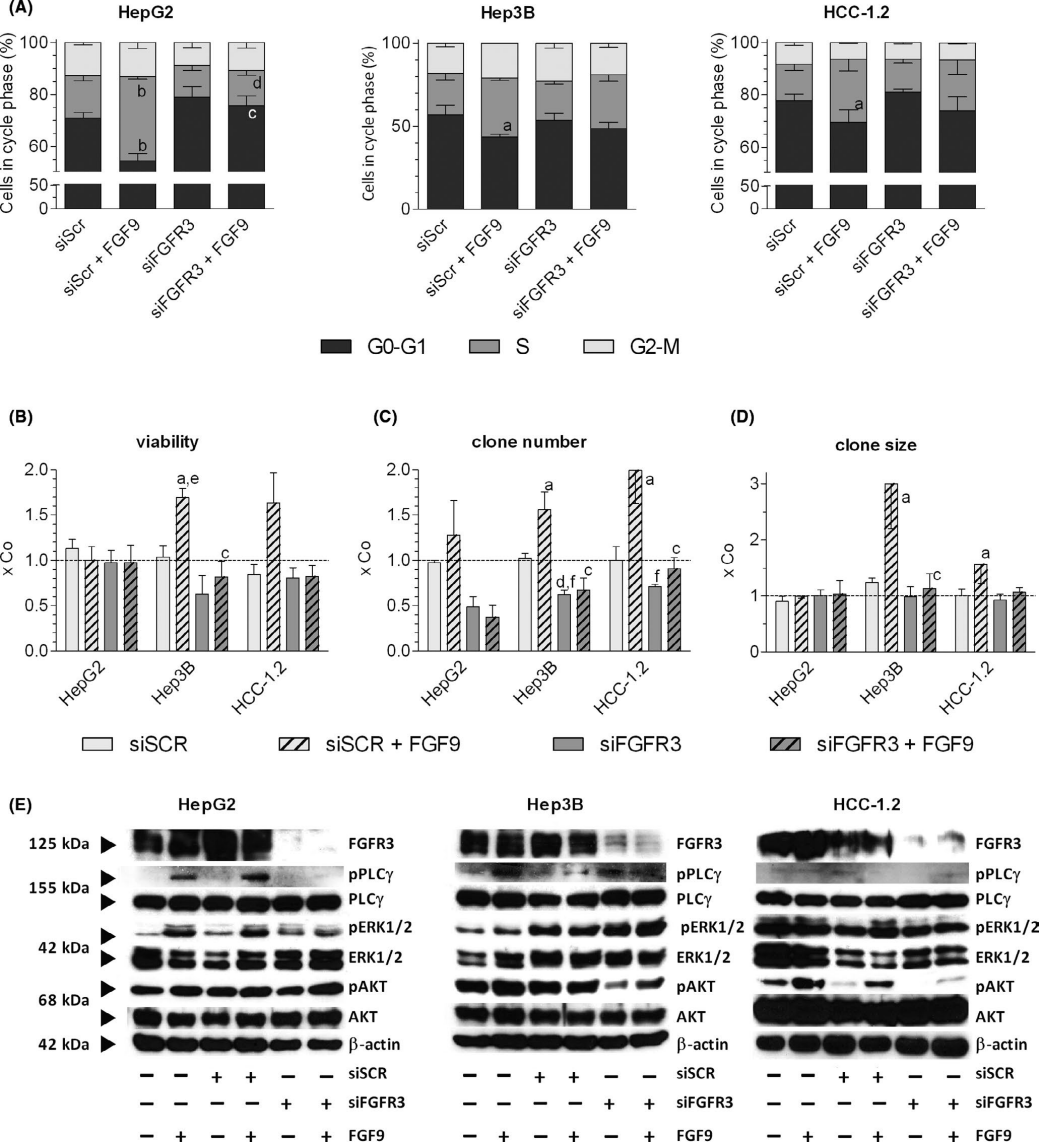
siFGFR3 antagonizes the FGF9‐mediated effects on cell cycle, viability, clone formation and signalling. (A‐D), Cells were transfected with siSCR or siFGFR3 (4392421/s5167, ThermoFisher Scientific) and re‐seeded in medium containing 1% FCS 24 h after transfection. Four hours later cells were treated with 10 ng FGF9/ml medium. Further methodical details see Table S6. (A), FACS determined the relative cell cycle distribution 48 h after FGF9 treatment. (B), Number of viable cells was determined by the MTT assay. (C‐D), Clones were fixed and stained with crystal violet after ~ 14 d. Number and size of clones were quantified by ‘LUCIA G image analysis software’. (E), 20 min after FGF9 stimulation, protein was isolated, separated on 10% SDS‐gels, and immunoblotted. (A‐D), Data are expressed as mean ± SEM of 3 independent experiments. Statistics by unpaired *t*‐test: any treatment without FGF9 vs any treatment with FGF9: a, *P* < .05; b, *P* < .01; any treatment without siFGFR3 vs any treatment with siFGFR3: c, *P* < .05; d, *P* < .01. Statistics by One Sample *t*‐test for any treatment vs control: e, *P* < .05; f, *P* < .01

siFGFR3 lowered transcript levels of both FGFR3 variants and the FGFR3 protein with high receptor specificity (Figure S8, Table S6).^7^ Considering the relatively high endogenous FGFR3 level in the cells, siFGFR3 alone reduced PLCγ phosphorylation, viability and percentage of cells in G0/G1/S phase, lowered clone formation at low density and in soft agar, and impaired gap‐forming capacity of the cells in BEC/LEC monolayers. In addition, the FGF9 induced increases in phosphorylation of downstream signalling components, viability of the cells and growth of clones were impaired completely by siFGFR3 (Figure 5,Table S6). This was strong evidence that FGF9 is a driver of the aggressive phenotype of hepatoma/hepatocarcinoma cells, mediated via FGFR3‐IIIb and/or FGFR3‐IIIc.

**FIGURE 6:**
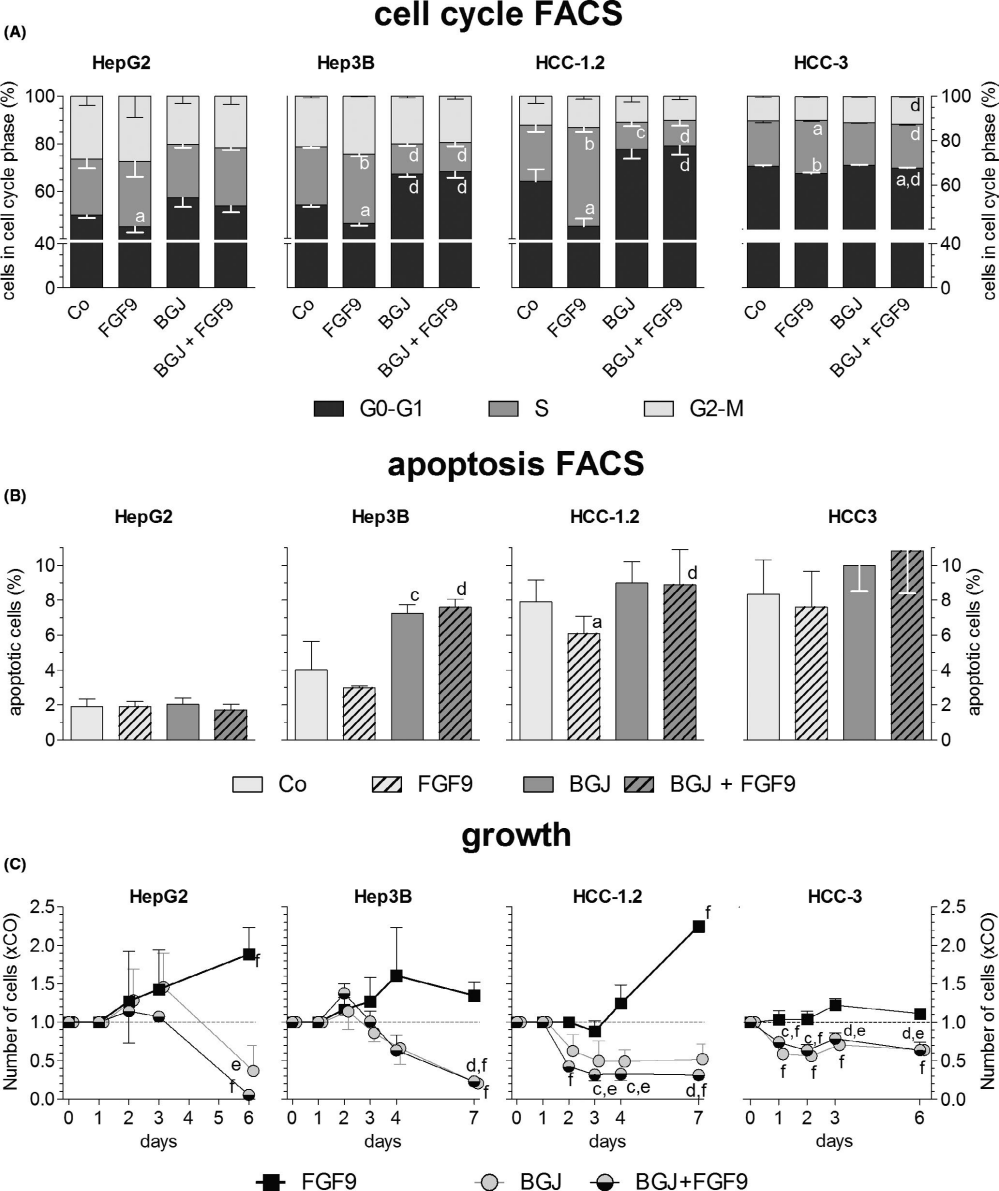
BGJ398 impairs FGF9‐induced growth by shifting cells to G0/G1 phase of cell cycle and/or to apoptosis. Twenty‐four h after seeding, hepatoma/hepatocarcinoma cells were treated with DMSO (Co) or BGJ398; 2 h later cells were stimulated with 10 ng FGF9/ml medium. Fourty‐eight h later, FACS determined the percentage of cells in the cell cycle phases (A) or undergoing apoptosis (B). (C), Cells were counted at regular intervals after start of FGF9 treatment (time point 0). (A‐C), Data are expressed as means ± SEM of 3 independent experiments. Statistics by unpaired *t*‐test: any treatment without FGF9 vs any treatment + FGF9: a, *P* < .05; b, *P* < .01; any treatment without BGJ vs any treatment + BGJ: c, *P* < .05; d, *P* < .01. Statistics by One‐Sample *t*‐test for any treatment vs control: e, *P* < .05; f, *P* < .01

**FIGURE 7:**
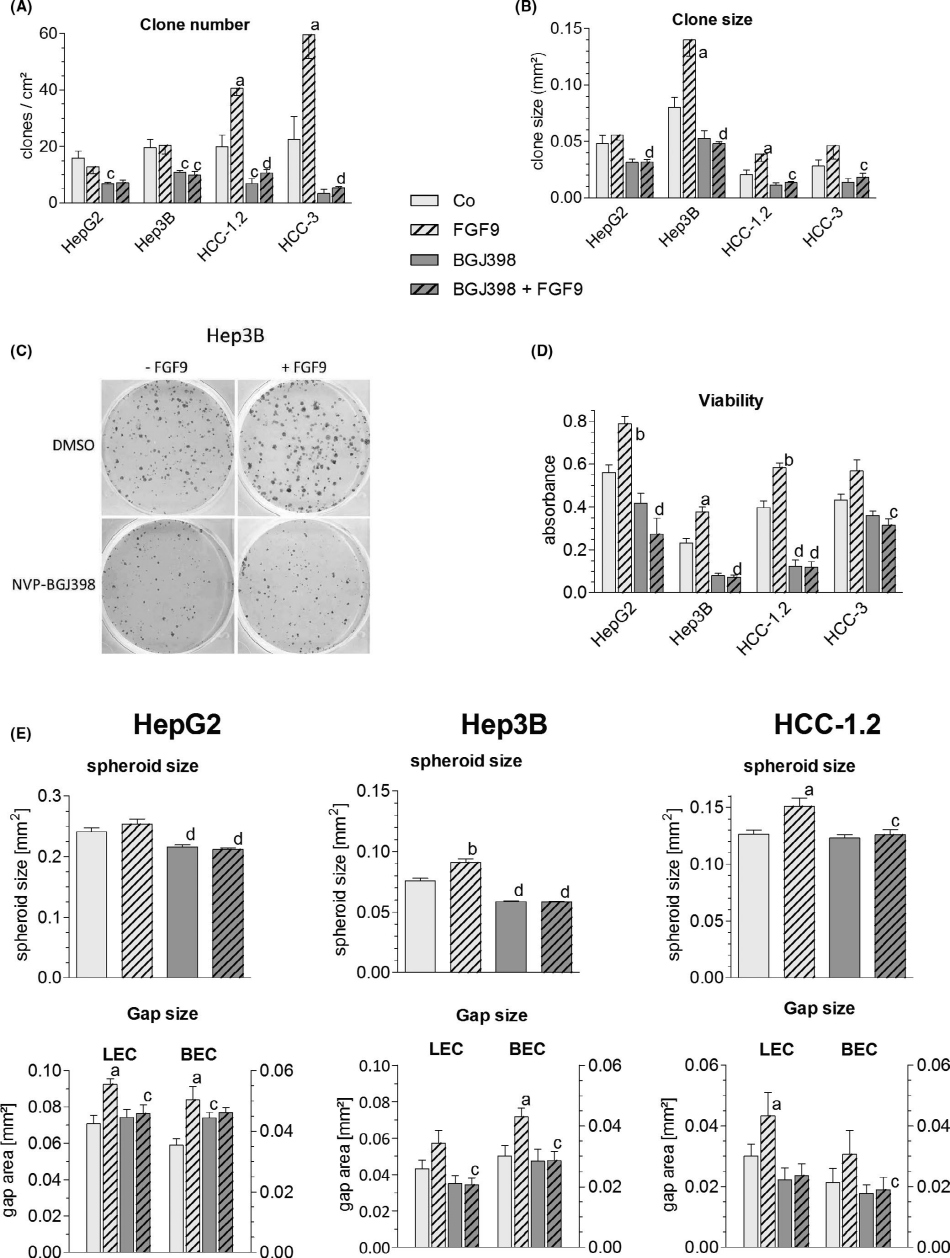
FGF9 enhances clonogenicity and invasiveness – blockade by BGJ398. (A‐D), Twenty‐four h after seeding hepatoma/hepatocarcinoma were treated with DMSO or BGJ398; 2 h later cells were stimulated with 10 ng FGF9/ml (hatched bars). (A‐C), Clones were fixed and stained with crystal violet after ~ 14 d. Number and size of clones were quantified by ‘LUCIA G image analysis software’. (C), shows representative images of Hep3B clones. (D), Number of viable cells was determined by the MTT assay. (E), Cells were used for the formation of spheroids, which were placed on BEC/LEC monolayers for gap formation. (A,B,D,E), Data are expressed as means ± SEM of ≥ 3 independent experiments. Statistics by unpaired *t*‐test: any treatment without FGF9 vs any treatment + FGF9: a, *P* < .05; b, *P* < .01; any treatment without BGJ vs any treatment + BGJ: c, *P* < .05; d, *P* < .01

BGJ398 halved the percentage of cells in the S‐phase, the clone‐forming capacity and cell viability in all cell lines tested (Figures 6A, 7A‐D). As a result, 7 days of treatment lowered the number of HepG2 and Hep3B cells by ~ 60% and arrested the growth of HCC‐1.2 and HCC‐3 cells (Figure 6C). BGJ398 blocked completely all FGF9‐mediated effects, including enhanced growth, increased proportion of cells in S‐phase, elevated clone size, phosphorylation of components of down‐stream signalling cascades as well as the migratory/invasive phenotype (Figures 6, 7, S10). Considering the lack of receptor specificity of BGJ398, the data from BGJ398 resemble those obtained by siFGFR3 which indicates that the effects of this tyrosine kinase inhibitor may be largely a result of FGFR3 blockade.

## 4 DISCUSSION

The present work provides novel insights into the role of the FGFR3‐IIIb/IIIc‐ligand FGF9 as potent growth factor enhancing the development and progression of HCC, as outlined in the following.

Structure analyses suggested that N‐ and C‐terminal regions of FGF9 are involved in homodimerization to support signalling.^15^, ^16^ Others proposed that homodimerization occludes receptor binding sites for auto‐inhibition and reduces monomer concentrations being sufficient to bind to IIIc‐but not to IIIb‐isoforms of FGFR1‐3.^17^ At 10 ng of FGF9 per ml medium (435 fmol) and a dissociation constant of 680 nM, FGF9 might have occurred primarily as monomers. Nevertheless, FGF9 elicited proliferation of hepatoma/hepatocarcinoma cells independent of their FGFR3‐splice variant profile, which could be blocked by knockdown of FGFR3 and not of the other FGFRs. This suggests that FGF9 acted preferentially on FGFR3‐IIIb and FGFR3‐IIIc at relatively low concentrations.

When compared to other FGFR3‐IIIb/IIIc ligands, FGF9 is the most efficient growth factor under our conditions and may act not only on human hepatoma/hepatocarcinoma cell lines but also on unaltered hepatocytes as well as on early stages of hepatocarcinogenesis (Figure S15). In unaltered liver, FGF9 appears to originate mainly from stellate cells and other mesenchymal liver cell types (Figure 3D, S7A) and to act on parenchymal hepatocytes. In chronic liver inflammation, stellate cells are activated to myofibroblasts which release FGF9.^18^ This might explain the somewhat increased FGF9 transcript levels in cirrhotic liver tissue (Figure 3A). Considering the strong growth inducing effect of FGF9 on preneoplastic hepatocytes, an elevated intrahepatic level of FGF9 in inflamed livers may promote the outgrowth of tumour prestages to frank malignancy via a paracrine mode of action. In contrast, in HCC FGF9 immunostainings were confined largely to the parenchyma with occasional occurrence of positive stroma cells (Figure 3E‐G). A similar pattern was seen when analysing parenchymal and mesenchymal cell lines, recently established from several HCC cases, i.e. relatively high FGF9 transcript levels in the hepatocarcinoma cells and a lower expression in the myofibroblasts (Figure S7C‐D). This is evidence that in advanced stages of hepatocarcinogenesis FGF9 may enhance growth and the aggressive phenotype by a predominantly autocrine mode of action.

The enhanced expression of FGF9 in a subset of HCC may be caused by deregulated miRNAs, as has been described for cells derived from HCC and other cancer entities.^19^, ^20^, ^21^ Upregulation of FGF9 in HCC may be caused also by hypoxia, e.g. in colon cancer cells, the FGF9 protein synthesis is repressed but switches to IRES‐dependent translation under hypoxic conditions.^22^ Considering that hypoxia may stimulate not only FGF9 protein synthesis but also neoangiogenesis, we tested whether FGF9 acts directly on endothelial cells in the liver. In fact, FGF9 elevated the replication of primary sinus endothelial cells and induced the differentiation of lymph‐ and hemangioendothelial cell lines to sprouts, tubes and nodes (bifurcations). An indirect positive effect on neoangiogenesis may be because of FGF9‐stimulated growth of myofibroblasts, a rich source of VEGF (Figure S4). The myofibroblasts derive from hepatic stellate cells and smooth muscle cells regulating the intrahepatic blood flow. These findings are reminiscent of FGF9 orchestrating the wrapping of vascular smooth muscle cells around newly formed vessels.^23^ Thus, FGF9 may contribute significantly to increases in the tumour mass by elevating not only proliferation of HCC cells but also by supporting neovascularization.

HCC generates early metastasis via invasion of tumour emboli (“bulky invasion”) into the blood or lymphatic circulation. To simulate this process, lymph‐ and hemangioendothelial monolayers were co‐incubated with spheroids of hepatoma/hepatocarcinoma cells. Within few hours gaps were formed in the monolayer mimicking entry/exit sites for intra‐ or extravasation. Under these experimental conditions FGF9 enhanced the invasive phenotype of all hepatoma/hepatocarcinoma cell lines by enhancing the disintegration of the endothelium by the spheroids. FGF9 induced further hallmarks of a prometastatic cell phenotype, i.e. the capability of migration and survival at low cell density (Figures 1, 4). These findings were supported by a clear trend to an infiltrative growth and a higher probability of recurrence in HCC cases when overexpressing FGF9 (Figure 3C) as well as by reports on models of various cancer entities.^19^, ^21^, ^24^, ^25^ To conclude, FGF9 may be an important driver of the aggressive phenotype by enhancing growth and invasiveness of HCC cells.

Considering the frequent co‐occurrence of FGF9 with FGFR3‐IIIb and/or FGFR3‐IIIc in HCC, we investigated whether FGF9 interacts mainly via FGFR3‐IIIb/IIIc. Almost complete antagonization of FGF9‐mediated effects became evident after knockdown of FGFR3, but not by silencing of FGFR1/2/4. Likewise, tyrosine kinase inhibition of FGFR1‐3 by BGJ398 could almost completely abolish all of the protumourigenic properties of FGF9. These findings imply that FGF9 acts mainly via FGFR3‐IIIb/IIIc in the hepatoma/hepatocarcinoma cells and that BGJ398 interferes with this interaction.

The present study suggests strongly that deregulations in the FGF9‐FGFR3‐IIIb/IIIc axis may be a potent target for the therapy of a subset of HCC. Different mechanisms may underlie the upregulated FGFR3 in about 50% of HCC cases, e.g. non‐coding FGFR3‐antisense transcripts may increase FGFR3 mRNA stability and expression in tumour cells.^26^ Higher FGFR3 protein levels occurred in 24% of HCC harbouring a FGFR3 gene with single nucleotide mutations in exon 9, 11 or 12.^27^ Every third HCC expresses a FGFR3 splice variant lacking partly Ig‐like‐III domain but binding FGF1/2 at normal levels.^28^ Another splice variant, found in every tenth HCC, lacks Ig‐like‐III domain and shows higher affinities for FGF1/2 than native FGFR3‐IIIc.^28^ These very recent findings indicate occurrence of receptor variants, modulating specificities and strengths of ligand‐FGFR3‐IIIb/IIIc interactions in HCC.

To conclude, multikinase inhibitors, interfering also with FGFR1‐FGFR4, improved somewhat overall survival and clinical outcomes of advanced‐stage HCC, indicating urgent needs to carefully select HCC patients on the base of suitable biomarkers.^3^ This study provides strong evidence that deregulations in the FGF9‐FGFR3‐IIIb/IIIc axis may serve to identify an HCC patients´ collective offering a potent therapeutic target.

## Conflict of Interest

All authors disclose any potential conflict.

## Supporting information

Supplementary Material
