# Supplementary material for: Interaction of FGF9 with FGFR3‐IIIb/IIIc, a putative driver of growth and aggressive behaviour of hepatocellular carcinoma
Source: Liver Int. 2020 Jun 17;40(9):2279–90. doi: 10.1111/liv.14505 (PMC7496895; doi:10.1111/liv.14505)
Supplement: Supplementary file 1 — Supplementary Material [file LIV-40-2279-s001.pdf]

SUPPORTING INFORMATION FOR:

Interaction of FGF9 with FGFR3-IIIb/IIIc, a putative driver of growth and aggressive behavior of hepatocellular carcinoma

Jakob Paur[1], Max Valler[1], Rebecca Sienel[1], Karin Taxauer[1], Klaus Holzmann[1], Brigitte Marian[1], Andreas Unterberger[1], Thomas Mohr[1], Walter Berger[1], Andja Gvozdenovich[1], Johannes Schimming[1], Michael Grusch[1], and Bettina Grasl-Kraupp[1]

[1] Department of Medicine I, Division: Institute of Cancer Research, Comprehensive Cancer Center Vienna, Medical University of Vienna, Borschkegasse 8a, 1090 Vienna, Austria

Table of content

Table S1. Age and sex of the patients, grade and size of HCC, and mRNA/protein expression levels of FGFR3-IIIb, FGFR3-IIIc and FGF9 in HCC and in non-cirrhotic control livers. p.4

Figure S1. FGF9 immunostaining controls. p.7

Table S2. Recombinant FGFs, antibodies, RT-qPCR assays and siRNAs applied. p.8

Table S3. Expression of FGFR3 splice variants in human hepatoma/hepatocarcinoma cell lines and in primary rat hepatocytes. p.10

Figure S2. FGF9 enhances the number of viable hepatoma /hepatocarcinoma cells. p.11

Table S4. FGF9 induces growth by shifting cells to S/G_2-M phase of cell cycle. p.12

Figure S3. FGF9 enhances cell migration – antagonism by BGJ398. p.13

Figure S4. FGF9 stimulates DNA replication of VEGF producing myofibroblasts. p.14

Figure S5. FGF9 upregulation in HCC – data from the “Genomic Data Commons Data Portal” p.15

Figure S6. Mesenchymal to epithelial switch of FGF9 expression in hepatocarcinogenesis. p.16

Figure S7. Predominant occurrence of FGF9 in mesenchymal cell types of unaltered rat liver and in epithelial cell lines, established from human HCC. p.17

Table S5. Expression levels of FGFR1, FGFR2, FGFR3, and FGFR4 in hepatoma/hepatocarcinoma cell lines and in primary rat hepatocytes. p.18

Figure S8. Effect of siRNA-mediated knock down on transcript and protein levels of FGFR1-4. p.19

Figure S9. Time-course of FGF9-induced phosphorylation of ERK1/2 and PLCy. p.20

Figure S10. FGF9 induces phosphorylation of PLCɣ and ERK1/2 – antagonism by BGJ398. p.21

Figure S11. Impact of FGFR1 or FGFR2 down-modulation on FGF9-mediated effects in hepatoma/hepatocarcinoma cells. p.22

Figure S12. Impact of FGFR4 down-modulation on FGF9-mediated effects in hepatoma/hepatocarcinoma cells. p.23

Figure S13. Cell lines with stable overexpression of FGFR3-IIIb or FGFR3-IIIc. p.24

Figure S14. FGF9 enhances clonal growth in hepatoma/hepato-carcinoma cells overexpressing FGFR3-IIIb. p.25

Table S6. Comparison of two siRNAs silencing FGFR3. p.26

Figure S15. FGF9 stimulates DNA replication of unaltered and initiated/premalignant rat hepatocytes. p.27

References p.28

***Table S1**. **Age and sex of the patients, grade and size of HCC, local infiltration, metastasis and/or recurrence, and mRNA/protein expression levels of FGFR3-IIIb, FGFR3-IIIc and FGF9 in HCC and in non-cirrhotic control livers.** The classification of the tumor grade followed the criteria published by Edmondson et al.[1] Abbreviations: m, male; f, female; +, local infiltration and/or occurrence of local/distant metastasis at the time point of resection of the primary tumor and/or tumor recurrence within a mean follow-up of 9±17 months; -, no local infiltration and/or occurrence of local/distant metastasis at the time point of resection of the primary tumor and/or no tumor recurrence within a mean follow-up period of 82±20 months; n.i., no information.*

A) HCC Cases									
Age	Sex	Grade of HCC	Size of HCC	Infiltr. Metas/ Recurr.	FGFR3-IIIb mRNA[a,b,d]	FGFR3-IIIc mRNA[a,b)]	FGFR3 Protein[b,c)]	FGF9 mRNA[a)]	FGF9 Protein[c)]
42	m	1	pT1	n.i.					
54	m	1	pT1	+					
74	m	1	pT1	n.i.					
66	m	1	pT1	-					
59	f	1	pT2	n.i.					
63	f	1	pT1	n.i.					
65	f	1	pT1	-					
69	f	1	pT1	+					
47	m	1	pT2	n.i.					
47	m	1	pT2	+					
50	m	2	pT1	+					
56	m	2	pT1	-					
56	m	2	pT1	-					
62	m	2	pT1	+					
66	m	2	pT1	-					
66	m	2	pT1	-					
67	m	2	pT1	-					
77	m	2	pT1	-					
77	m	2	pT1	-					
78	m	2	pT1	-					
38	f	2	pT1	-					
77	f	2	pT1	+					
82	f	2	pT1	-					
48	m	2	pT2	-					
59	m	2	pT2	-					
67	m	2	pT2	-					
67	m	2	pT2	-					
67	m	2	pT2	n.i.					
69	m	2	pT2	-					
69	m	2	pT2	+					
76	m	2	pT2	+					
61	f	2	pT2	+					
65	f	2	pT2	-					
22	m	2	pT3	n.i.					
34	m	2	pT3	n.i.					

49	m	2	pT3	n.i.					
64	m	2	pT3	n.i.					
68	m	2	pT3	n.i.					
70	m	2	pT3	+					
59	f	2	pT3	n.i.					
65	f	2	pT3	n.i.					
70	f	2	pT3	-					
77	f	2	pT3	n.i.					
49	m	2	pT4	n.i.					
53	m	2	pT4	n.i.					
55	m	2	pT4	+					
57	m	2	pT4	n.i.					
63	m	2	pT4	n.i.					
67	m	2	pT4	-					
63	m	2	n.d.	+					
43	f	2	pT4	+					
71	f	2	pT4	+					
55	m	3	pT2	n.i.					
68	m	3	pT2	+					
78	f	3	pT2	-					
48	m	3	pT3	+					
58	m	3	pT4	n.i.					
77	f	3	pT4	n.i.					

B) Control Livers without Cirrhosis

Age	*Sex*	*Liver Alterations*	*FGF9 mRNA Expression (absolute, ΔCt)* a)
55	f	Carcinoma of gallbladder	17.0
64	f	Metastasis of colon-adenocarcinoma	15.5
75	m	Pseudo-tumor	14.6
77	m	Metastasis of colon-adenocarcinoma	14.3
53	f	Echinococcus cyst	14.0
72	m	Metastasis of colon-adenocarcinoma	13.7
54	f	Metastasis of colon-adenocarcinoma	13.4
53	f	Echinococcus cyst	13.3
72	f	Metastasis of colon-adenocarcinoma	13.0
70	f	Metastasis of colon-adenocarcinoma	11.5

a) Messages were quantified by RT-qPCR, which was performed on an ABI PRISM 7500 system (Applied Biosystems, Foster City, CA) using assay kits (Applied Biosystems) or custom-made primers (see Table S2). The number of amplification cycles for the fluorescent reporter signal of the gene of interest to reach a common threshold value (Ct-value) was estimated by using the ABI-PRISM 7500 software. The Ct-value of the gene of interest was normalized by subtracting the Ct-value obtained from the same sample for ß-actin (ΔCT-value). The expression level in HCC, relative to that in the surrounding liver tissue, is expressed as $2{-}\Delta\Delta Ct$, which reflects ΔCt-value of the HCC minus ΔCt-value of the surrounding non-tumorous liver tissue (normalized to 1).

b) Please note that the expression data on FGFR3 have been published previously.[2]

c) Immunostaining: For the validation of FGF9 antibodies see Figure S1. The immunostaining for FGFR3 and FGF9 of each tumor was compared to the surrounding tissue (ST). The extent of staining was estimated by the percentage of cells of a certain staining intensity. The intensity of staining within the tumor was categorized on an arbitrary scale of 0 to 3, i.e., 0, no immunoreaction; 0.5, weaker immunoreaction than in ST; 1, equal to ST; 2, somewhat stronger than in ST; 3, much stronger than in

ST. To avoid intra-observer variability all slides were evaluated in a blinded fashion by the same person. In every case the appraisal was performed in five representative microscope fields per tumor and per ST (magnification x50). Intensity and extent of staining served to calculate a score for the expression level; e.g. 20% of the cells were grouped into staining category 0.5 and 80% of the cells into category 2 (0.2 x 0.5 = 0.1; 0.8 x 2 = 1.6; 0.1 + 1.6 = 1.7); the score for the expression level was calculated to be 1.7. The ratio between the score of the tumor and the score of the surrounding was calculated for each sample: a value of < 1 indicates less protein in the tumor than in the surrounding, 1 equal to, and > 1 indicates more protein in the tumor.

d) Color code in (A):

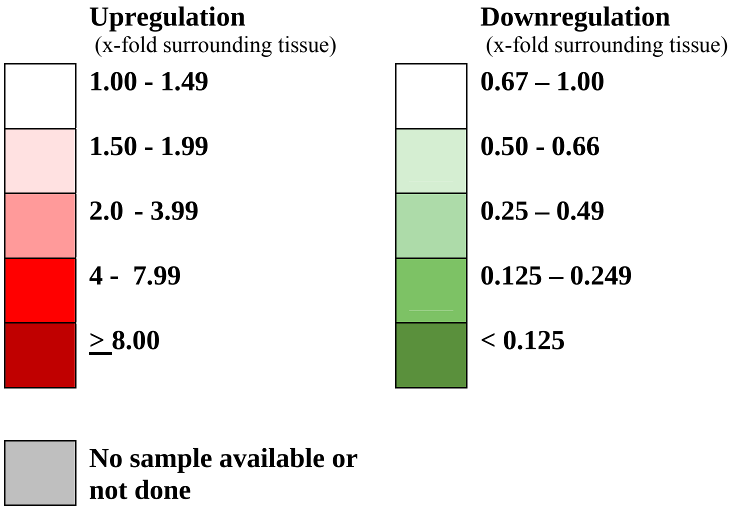

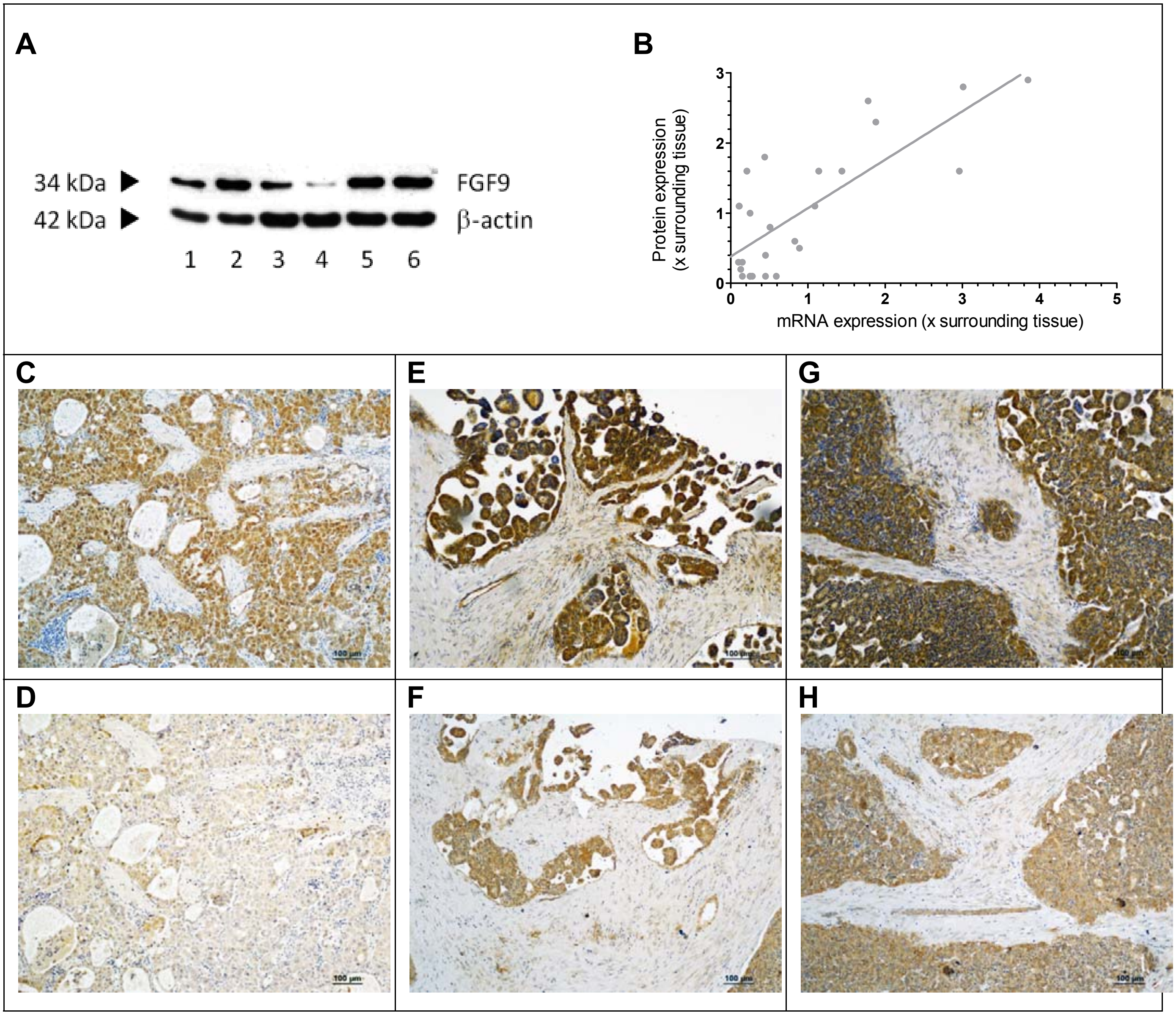

Figure S1. FGF9 immunostaining controls. *Two antibodies were tested, which had been raised against full-length human recombinant FGF9 (Met1-Ser208; accession number at NCBI # P31371): mab-273, a monoclonal mouse IgG2A (clone # 3691; R&D) and sc-7876, a rabbit polyclonal antibody (Santa-Cruz Biotechnology). (A), Human hepatoma/hepatocacinoma cells were kept under standard culture conditions. Protein was isolated, separated on 10% SDS-gels, and immunoblotted, following published protocols.[2] The blots were probed with sc-7876, staining specifically a band occurring at the molecular weight of FGF9. Bands1-2, HepG2; bands 3-4, Hep3B; bands 5-6, HCC-1.2. (B), Correlation between extent of immunostaining and expression of FGF9 at the mRNA level in HCC, when compared to the surrounding tissue: r2 = 0.6085; p < 0.01. Further details see Table S1. (C-H), Sections, obtained from formalin-fixed and paraffin-embedded tissue, were stained by immunohstochemistry, as described in detail before.[2] HCC of tumor grade 2 stained with sc-7876 (C) and negative control without primary antibody (D); HCC of tumor grade 3 stained with sc-7876 (E) and mab273 (F); HCC of tumor grade 1, stained with sc-7876 (G) and mab-273 (H).*

Table S2. Recombinant FGFs, antibodies, RT-qPCR assays and siRNAs applied.

Recombinant protein	
**FGF1**	BioVision, Milpitas, CA
**FGF2**	BioVision, Milpitas, CA
**FGF4**	BioVision, Milpitas, CA
**FGF8**	BioVision, Milpitas, CA
**FGF9**	R&D System, Minneapolis, MN
**FGF17**	BioSource, San Diego, CA
**FGF18**	BioVision, Milpitas, CA
**Vascular endothelial growth factor** (VEGF, 354107)	Corning
Primary antisera for immunodetection	
**Anti-β-actin** (AC-15) (ab6276)	Abcam, Cambridge, UK
**Anti-AKT (pan)** (C67E7) (# 4691)	Cell Signaling, Danvers, MA
**Anti-ERK 1/2**	Sigma, St Louis, MO
**Anti-FGF9** (AF-273-NA)	R&D Systems, Minneapolis, MN
**Anti FGF9**	Santa Cruz, Dallas, TX
**Anti-cd34** (ab81289)	Abcam, Cambridge UK
**Anti alpha-smooth muscle actin** (M0851)	Dako, Santa Clara, CA
**Anti-FGFR3** (C-15): sc-123	Santa Cruz, Dallas, TX
**Anti-GSTp** (anti-rat Yp subunit of placental glutathione-S-transferase)	Biotrin-International, Dublin, Eire
**Anti-phospho-AKT** (Ser437) (# 9271s)	Cell Signaling, Danvers, MA
**Anti-phospho-p44/42 MAPK (ERK 1/2)** (Thr202/Tyr204) (# 9101)	Cell Signaling, Danvers, MA
**Anti-phospho-PLCγ1** (Tyr783) (# 2821)	Cell Signaling, Danvers, MA
**Anti-PLCγ1** (# 2822)	Cell Signaling, Danvers, MA
Secondary antisera for immunodetection	
**Horseraddish peroxidase (HRP)-labeled anti-rabbit IgG**	Bethyl Laboratories, Inc., Montgomery, TX
**Polyclonal goat Anti-mouse IgG – HRP** (P0447)	Dako, Santa Clara, CA
**Polyclonal goat Anti-rabbit IgG – HRP** (P0448)	Dako, Santa Clara, CA
Taqman assays and primers for qRT-PCR	
**Human**	
**ß-actin** (custom-made)	Eurofins, Vienna, Austria
**FGFR1** (Hs00915137_m1)	Applied Biosystems, Foster City, CA
**FGFR2** (Hs01552926_m1)	Applied Biosystems, Foster City, CA
**FGFR3** (Hs00179829_m1)	Applied Biosystems, Foster City, CA
**FGFR3-IIIb** (Hs01005396_m1)	Applied Biosystems, Foster City, CA
**FGFR3-IIIc** (Hs00997397_m1)	Applied Biosystems, Foster City, CA
**FGFR4** (Hs01107438_m1)	Applied Biosystems, Foster City, CA
**FGF9** (Hs00181829_m1)	Applied Biosystems, Foster City, CA
**Rat**	
**ß2-microglobulin** (Rn00560865_m1)	Applied Biosystems, Foster City, CA
**FGFR1** (Rn00577234_m1)	Applied Biosystems, Foster City, CA
**FGFR2 forward** (custom-made) 5´-CATCGGAGGCTATAAGGTACGAA-3´	TIB Molbiol, Berlin, FRG
**FGFR2 reverse** (custom-made) 5´-CAGGTGTAATTGCCTTTGTCTGAT-3´	TIB Molbiol, Berlin, FRG
**FGFR3** (Rn00584799_m1)	Applied Biosystems, Foster City, CA
**FGFR3-IIIb forward** (custom-made) 5'-AGCTGCAAACACTGTACG-3'	Eurofins, Vienna, Austria
**FGFR3-IIIb reverse** (custom-made) 5'-CCGGATGCTGCCAAACTT-3'	Eurofins, Vienna, Austria
**FGFR3-IIIc forward** (custom-made) 5'-AGCTGCAAACACTGTACG-3	Eurofins, Vienna, Austria
**FGFR3-IIIc reverse** (custom-made) 5'-CAAAGGTGACATTGTGCAA-3'	Eurofins, Vienna, Austria
**FGFR4 forward** (custom-made) 5´-ACTCAGGAAGGGCCCCTGTAT-3´	MWG Biotech, Ebersberg , FRG
**FGFR4 reverse** (custom-made) 5´-CCGGGCACGGAGGAA-3´	MWG Biotech, Ebersberg , FRG
siRNA	

**siFGFR1** (#4390824/s5164)	Thermo Fisher Scientific, Waltham, MA
**siFGFR2** (#4392420/s5173)	Thermo Fisher Scientific, Waltham, MA
**siFGFR3** (#4392421/s5167)	Thermo Fisher Scientific, Waltham, MA
**siFGFR3** (#AM16708/ assay ID 110728)	Thermo Fisher Scientific, Waltham, MA
**siFGFR4** (#4390824)	Thermo Fisher Scientific, Waltham, MA
**Scrambled siRNA** (AM4390843)	Thermo Fisher Scientific, Waltham, MA

Table S3. Expression of FGFR3 splice variants in human hepatoma/hepatocarcinoma cell-lines and in primary rat hepatocytes. *Primary hepatocytes were isolated from rat liver by collagenase perfusion, as described;[3] mRNA was isolated from cells and of hepatoma/hepatocarcinoma cell lines; expression levels of FGFR3-splice variants were determined by RT-qPCR. Further details see Table S1. Data are ΔCT-values, presented as mean + SD from >2 independent cell preparations or single determinations.*

	**FGFR3-IIIb**	**FGFR3-IIIc**
**Human hepatoma/hepatocarcinoma cell lines**		
HCC-1.1	17.9[a]	16.3
HCC-1.2	6.8 + 0.3	5.1 + 0.9
HCC-2	8.7	11.0
HCC-3	7.7 + 0.3	5.9 + 1.2
HepG2	3.3 + 0.7	4.0 + 0.1
Hep3B	5.1 + 0.3	8.1 + 0.5
**Primary rat hepatocytes**		
	6.3 + 0.3	13.7 + 0.4

[a] *Please note that an increase in ΔCT-value by 1.0 indicates a twofold decrease in the gene expression level.*

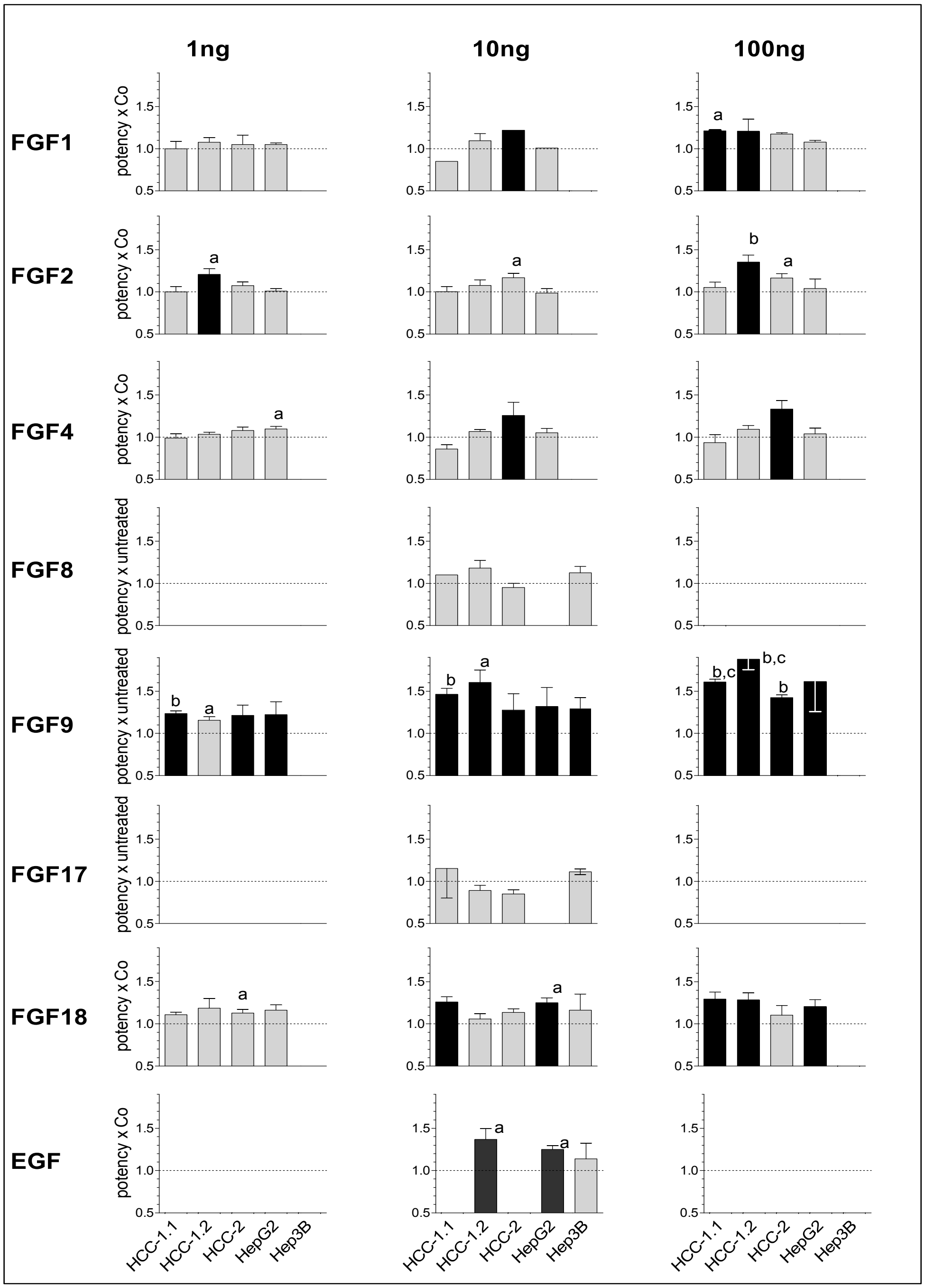

Figure S2. FGF9 enhances the number of viable hepatoma/hepatocarcinoma cells. *Cell lines were serum-starved for 48hrs, followed by treatment with 1, 10 or 100ng of FGF/ml medium <u>or</u> 10ng EGF/ml medium for 72hrs. The number of viable cells was determined with the EZ4U assay (see Methods). Each bar represents a dataset of one of the 5 cell lines tested and shows the mean of fold control values obtained from >3 independent experiments. Black bars indicate that the mean value was elevated >1.2 above the control level. SEMs are given. Please note that the growth inducing potential of FGF8, 17 and 18 was reported by Gauglhofer et al 2011.[4] Statistical analyses were performed with the One-Sample t-test: a, $p < 0.05$; b, $p < 0.01$, and the Kruskal-Wallis test (over concentration range) c, $p < 0.05$.*

Table S4. FGF9 induces growth by shifting cells to S/G_2-M phase of cell cycle. *Twenty-six hrs after seeding cells were treated with 10ng FGF9/ml medium. Fourty-eight hrs later FACS determined the percentage of cells in the cell cycle phases or undergoing apoptosis. Further experimental details are given in Methods. Data are expressed as means ± SEM of $\geq$3 independent experiments. Statistics by unpaired t-test for Co vs. FGF9: a, $p<0.05$; b, $p<0.01$.*

		% of Cells in **Go/G1-Phase**	% of Cells in **S-Phase**	% of Cells in **G2/M-Phase**	% of **Apoptosis**
**HepG2**	Co	50.1 ± 1.1	26.6 ± 3.9	23.3 ± 4.0	1.9 ± 0.5
	FGF9	46.4 ± 1.8 [a]	28.1 ± 3.9 [a]	25.5 ± 5.6 [a]	1.9 ± 0.3
**Hep3B**	Co	54.1 ± 0.7	24.5 ± 0.2	21.4 ± 0.6	4.0 ± 1.0
	FGF9	48.0 ± 1.7[a]	28.9 ± 0.8[b]	23.1 ± 1.2	2.9 ± 0.1[a]
**HCC-1.2**	Co	64.4 ± 5.0	23.3 ± 2.7	12.3 ± 2.9	7.9 ± 0.6
	FGF9	45.6 ± 3.1[a]	41.3 ± 1.9[b]	13.1 ± 1.3	6.1 ± 0.5 [a]
**HCC-3**	Co	68.4 ± 0.6	20.5 ± 0.8	11.1 ± 0.5	8.4 ± 1.0
	FGF9	65.3 ± 0.3[b]	23.8 ± 0.4[a]	10.9 ± 0.3	7.6 ± 1.0

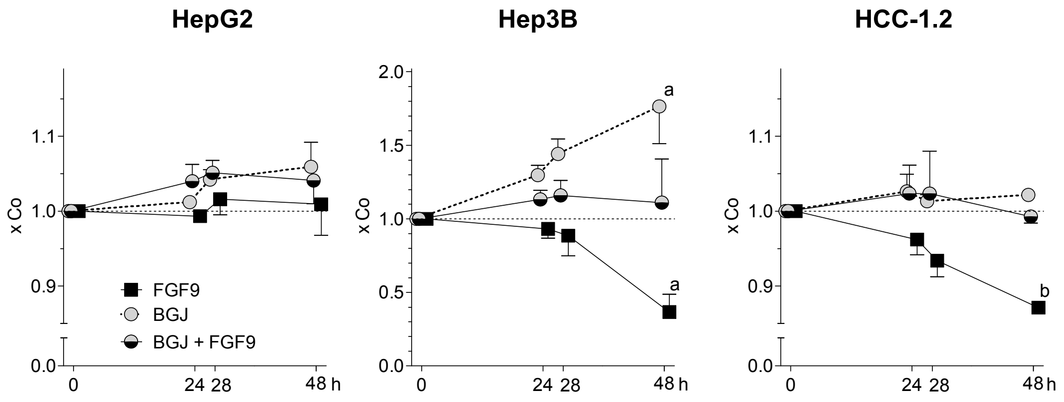

Figure S3. FGF9 enhances cell migration – antagonism by BGJ398. *Confluent monolayers of heptoma/hepatocarcinoma cells were switched to serum-free medium and were scratched by using a sterile 200µl pipette tip. Cells were rinsed and subsequently treated with BGJ398 at 1mM (HepG2) or 500uM (Hep3B, HCC-1.2). Two hrs later, FGF9 was added at 10ng/ml medium. Total area of the scratches was measured by Image-J software on day 0 (day of treatment) and after 24, 28 and 48 hrs of treatment. Data are expressed as mean ± SD of fold Co (DMSO) of 3 independent experiments. Statistics by One-Sample t-test for control vs treatment: a, $p < 0.05$; b, $p < 0.01$.*

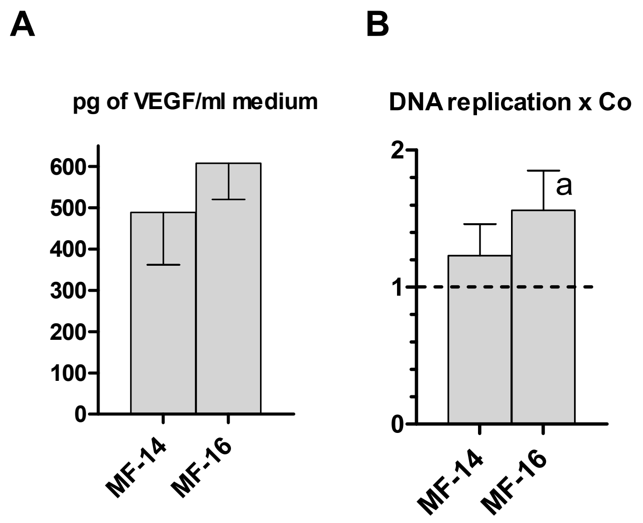

Figure S4. FGF9 stimulates DNA replication of VEGF producing myofibroblasts. *The myofibroblast cell lines (MF-14, MF-16) were recently established from 2 HCC cases and characterized.[5] (A), In conditioned media (72hrs of culture) VEGF was determined by ELISA according to manufacturer´s instructions (R&D Systems, Abingdon, UK). (B), Cells were kept under standard conditions and treated with 10ng of FGF9 per ml medium for 48 hrs. DNA replication was determined by scintillation counting, as described (5). (A,B), Data are means ± SD of 3 independent experiments. Statistics by One-Sample t-test: a, $p < 0.05$.*

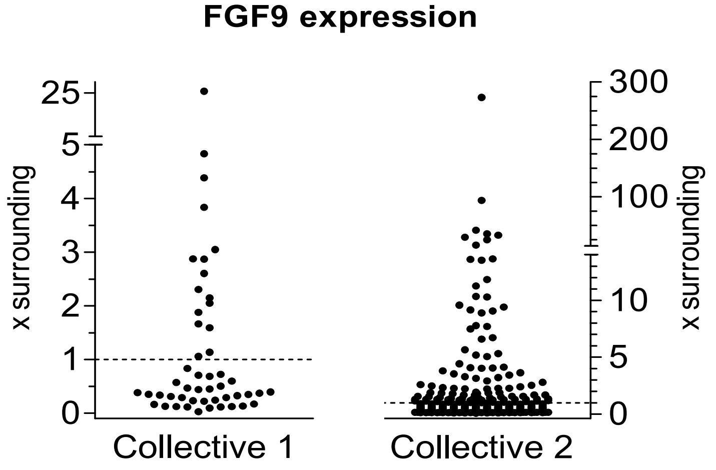

Figure S5. FGF9 upregulation in HCC – data from the "Genomic Data Commons Data Portal". *Raw data were retrieved from the "Genomic Data Commons Data Portal" of the National Cancer Institute (https://portal.gdc.cancer.gov), normalized by TMM (trimmed mean of m-values) and transformed by Voom. In 49 cases FGF9 expression levels were provided for HCC tissue samples and matched non-tumor liver tissue (collective 1); for 355 cases information was available for HCC tissue only (collective 2). For collective 1 in the left panel, FGF9 transcript levels in individual HCC were normalized by the matched non-tumor liver tissue and are expressed as x surrounding tissue. Collective 2 in the right panel shows the FGF9 expression in individual HCC, normalized by the median of the FGF9 levels obtained from non-tumor liver tissue of collective 1; data are expressed x surrounding of collective 1. Values >1 indicate a higher FGF9 transcript level in HCC than in non-tumor liver tissue. Statistical analysis by Student´s t test: no significant difference was found between collective 1 and 2 supporting the validity of the mathematical approach.*

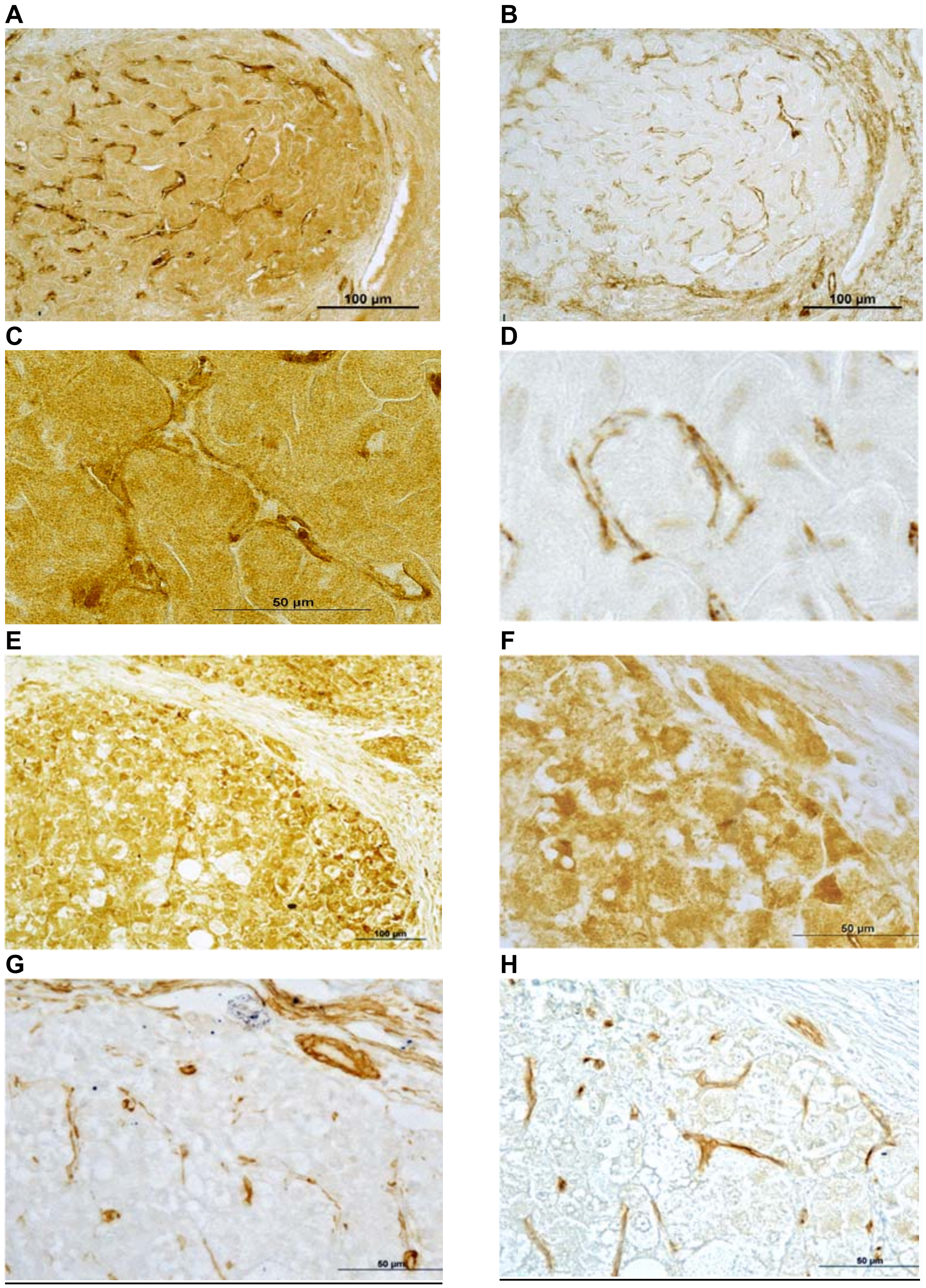

Figure S6. Mesenchymal to epithelial switch of FGF9 expression in hepatocarcinogenesis. *Serial sections, obtained from formalin-fixed and paraffin-embedded liver tissue, were stained by immunohistochemistry for FGF9, α-smooth muscle actin (a marker for activated stellate cells and pericytes), and cd34 (a marker for blood endothelial cells). Cirrhotic liver stained for FGF9 (A,C) and α-smooth muscle actin (B,D). HCC of tumor grade 1 stained for FGF9 (E, F), α-smooth muscle actin (G) and cd34 (H)*

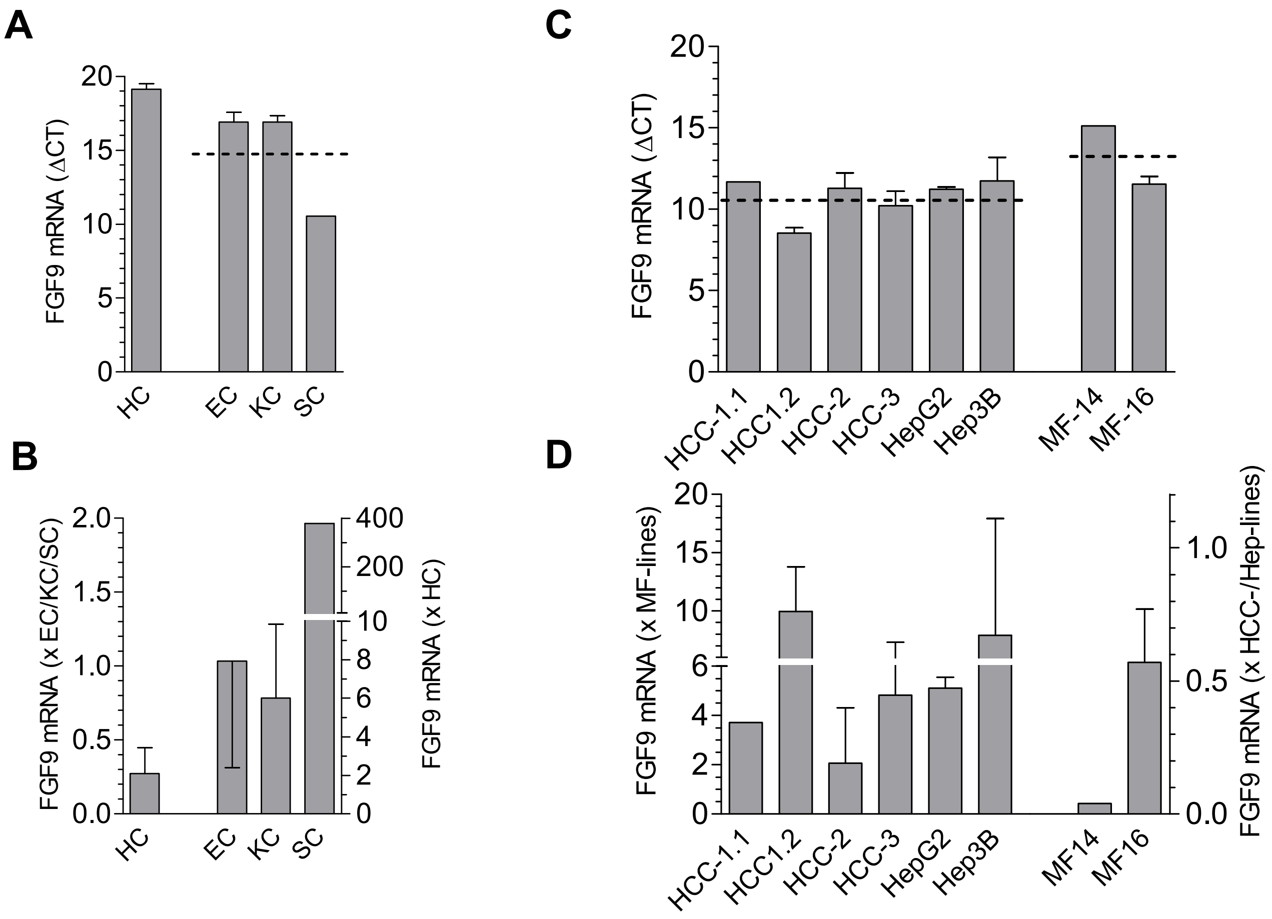

Figure S7. Predominant occurrence of FGF9 in mesenchymal cell types of unaltered rat liver and in epithelial cell lines, established from human HCC. *(A) Livers of male Wistar rats were perfused with collagenase. The cell suspension obtained was used to separate hepatocytes (HC) from mesenchymal cells by low speed centrifugation in percoll gradients. Mesenchymal cells were further separated into endothelial cell (EC)-, Kupffer cell (KC)-, and stellate cells (SC)-enriched fractions, as described.*[3] *(C), mRNA was isolated from hepatoma/hepatocarcinoma cell lines (HCC-1.1, HCC-1.2, HCC-2, HCC-3, HepG2, Hep3B) and from myofibroblast cell lines (MF-14, MF-16). Further details on cell lines see Methods. (A, C), The expression levels of FGF9 were determined by RT-qPCR. Please note that a decrease in ΔCt by 1.0 indicates a twofold increase in the gene expression level. The dashed lines indicate the means. (B), The FGF9 expression in HC is calculated as fold of the level found in the mesenchymal cells (EC, KC, SC) and vice versa. (D), the FGF9 expression in hepatoma/hepatocarcinoma lines is shown as fold of the level found in myofibroblast cell lines (MF-lines) and vice versa. (A-D), The data are expressed as means ± SD from 2-3 independent cell preparations.*

Table S5. Expression levels of FGFR1, FGFR2, FGFR3, and FGFR4 in hepatoma/hepatocarcinoma cell lines and in primary rat hepatocytes. *Primary hepatocytes were isolated from rat liver by collagenase perfusion, as described [3]; mRNA was isolated from hepatocytes and hepatoma/hepatocarcinoma cell lines. Expression levels of FGFRs were determined by RT-qPCR. Further details see Table S1. Data are ΔCT-values, presented as mean ± SD from ≥2 independent cell preparations or single determinations.*

	**FGFR1**	**FGFR2**	**FGFR3**	**FGFR4**
**Human hepatoma/hepatocarcinoma cell lines**				
HCC-1.1	7.4 ± 0.8[a]	6.5 ± 0.5	15.4 ± 7.2	7.1 ± 0.5
HCC-1.2	10.8 ± 1.5	11.0 ± 0.3	6.6 ± 0.6	6.4 ± 0.3
HCC-2	9.0 ± 1.3	3.9 ± 0.3	7.2 ± 5.6	1.3 ± 0.2
HCC-3	8.2	11.2	7.3 + 0.2	5.0
HepG2	4.8 ± 1.7	9.0 ± 0.1	4.2 ± 0.6	4.1 ± 0.2
Hep3B	6.2 ± 0.1	7.6 ± 0.1	6.0 ± 0.6	6.1
**Primary rat hepatocytes**				
	14.0 ± 0.3	8.7 ± 0.3	8.3 ± 0.1	7.3 ± 0.2

[a] *Please note that an increase in ΔCT-value by 1.0 indicates a twofold decrease in the gene expression level.*

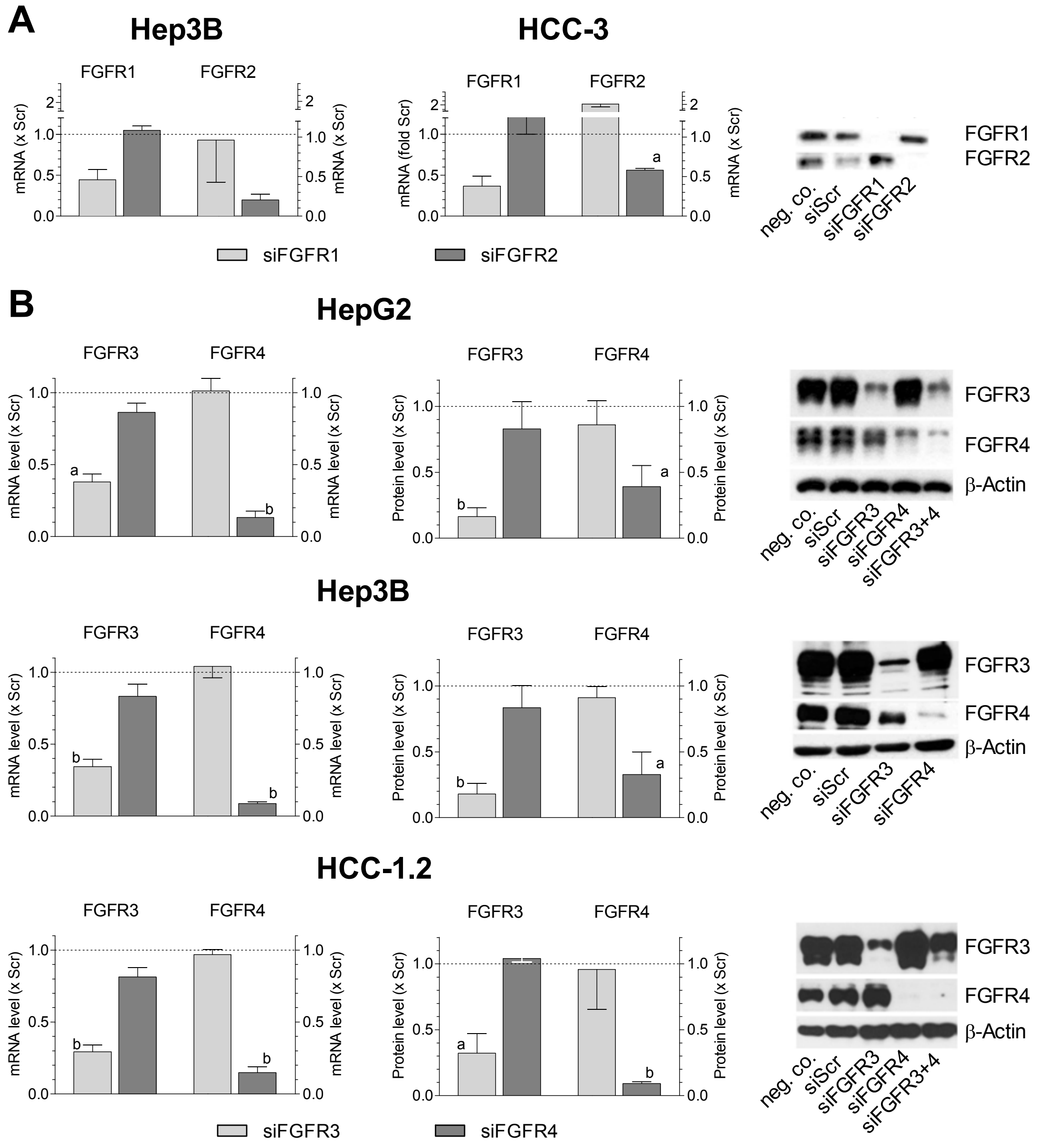

Figure S8. Effect of siRNA-mediated knock down on transcript and protein levels of FGFR1-4. *Twenty-four hrs after seeding, cells were transfected with FGFR-specific siRNAs (see Table S2). scrambled siRNA (siScr) was used as transfection control. Forty-eight hrs later cells were harvested and mRNA and protein were isolated. Transcripts of FGFR1-4 were determined by RT-qPCR. Total protein of cells was separated by gel electrophoresis, analysed by western blot, and subjected to densitometry. (A,B), Data are expressed as fold siScr and are means ± SEM of* $\geq$*2 independent experiments. One-Sample t-test was performed to determine the significance for siScrl vs. FGFR-specific siRNA: a, p < 0.05; b, p<0.01.*

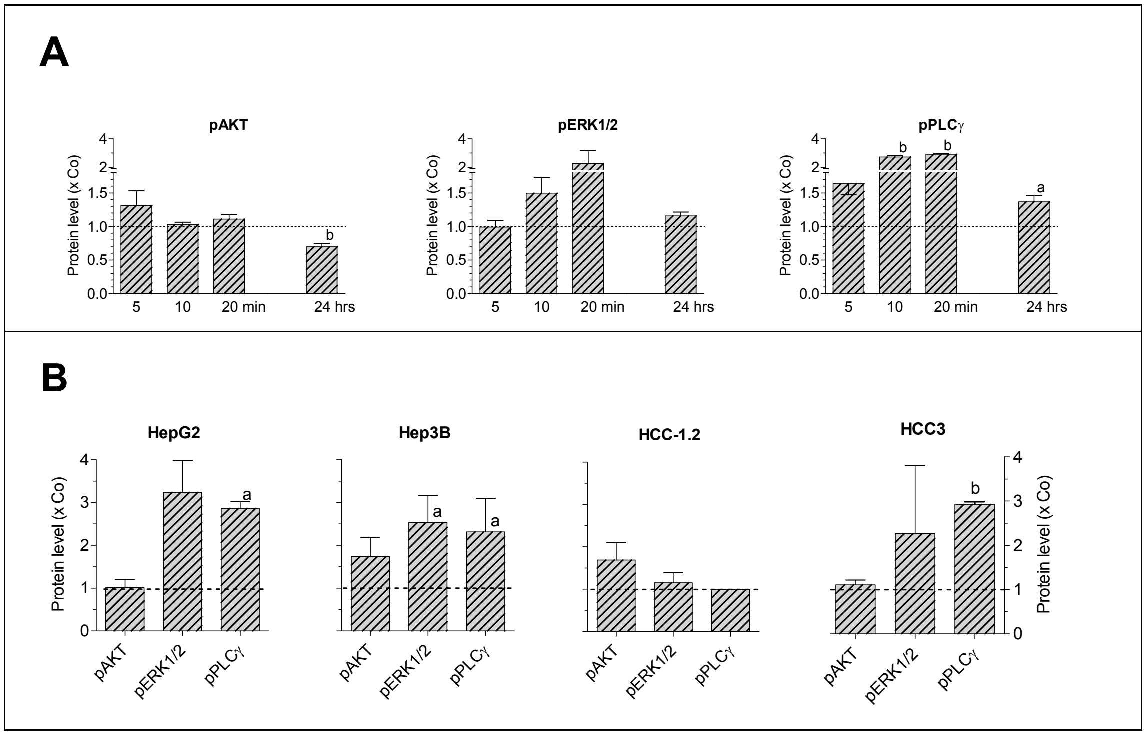

Figure S9. Time-course of FGF9-induced phosphorylation of AKT, ERK1/2 and PLCy. *Twenty-fours hrs after seeding, cells were treated with 10ng of FGF9/ml medium. Five, 10 and 20 min and 24hrs later, cells were harvested, as shown for HCC-3 in (A). Similar time-course studies were run with the other cell lines to determine the optimal time point of investigation (not shown). (A, B) Thirty ng of protein were separated by gel electrophoresis in 10% SDS-gels and analysed by western blot; ß-actin was used as housekeeping protein (Co). Band intensities were quantified by using Image J software for densitometry. Data are means ± SEM of 2-5 independent experiments. Statistics by One sample t-test a, p<0.05; b, p< 0.01.*

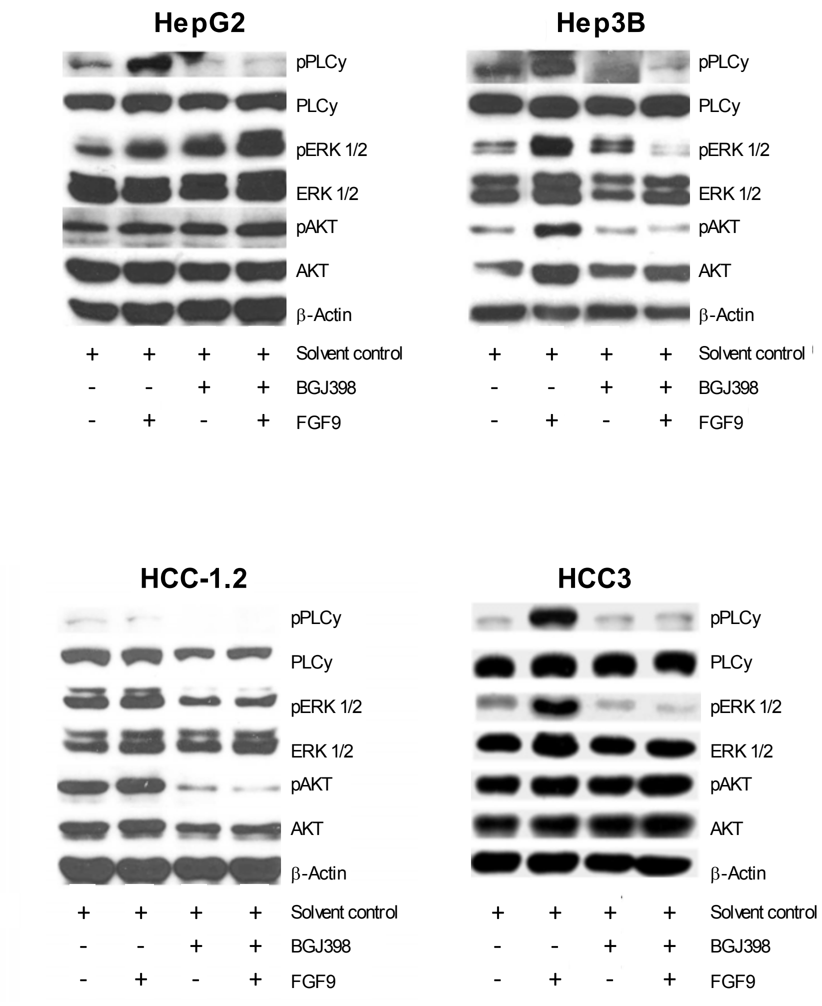

Figure S10. FGF9 induces phosphorylation of PLCɣ and ERK1/2 – antagonism by BGJ398. *Twenty-four hrs after seeding cells were treated either with DMSO (solvent control) or BGJ398 (dissolved in DMSO); 2 hrs later, half of the cell culture dishes of each treatment group were stimulated with 10ng of FGF9 per ml medium; 20min later cells were harvested and proteins were isolated; 30ng of proteins were separated on 10% SDS-gels and analyzed by western blot.*

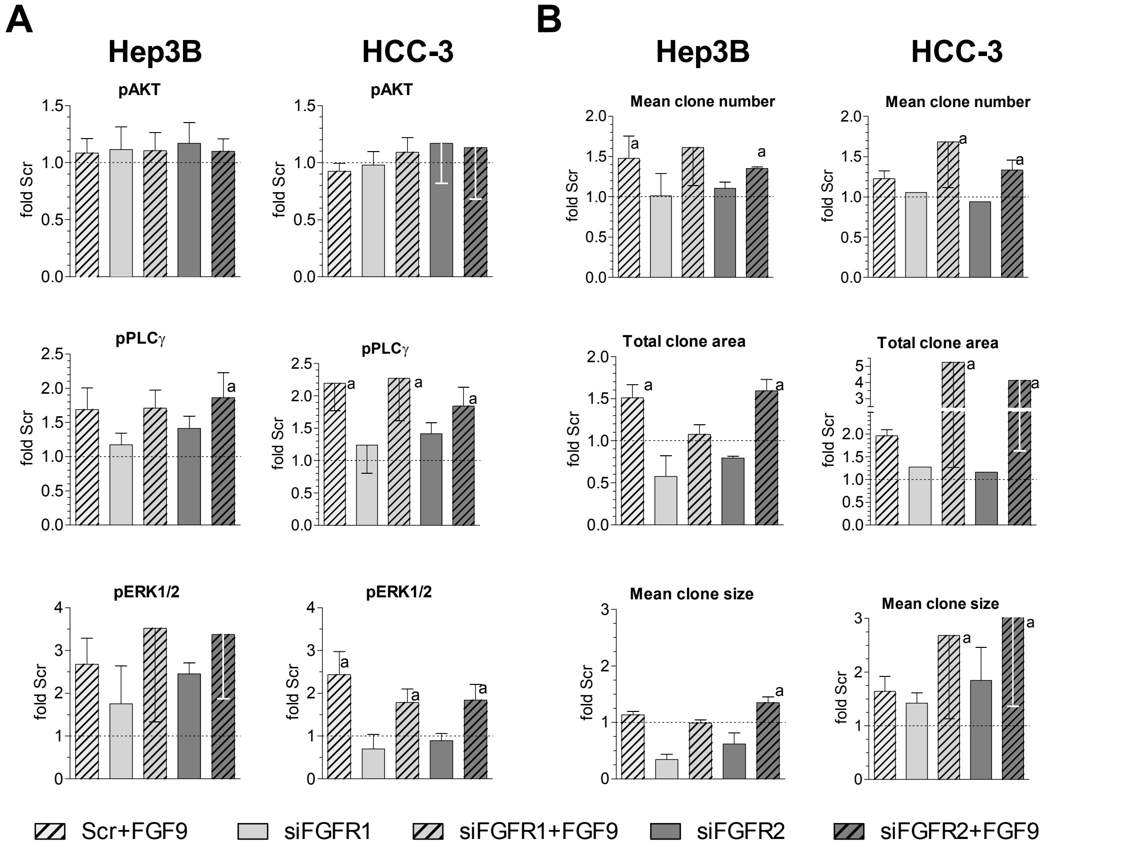

Figure S11. Impact of FGFR1 or FGFR2 down-modulation on FGF9-mediated effects in hepatoma/hepatocarcinoma cells. *Hep3B and HCC-3 cells were transfected with siRNA against FGFR1 (siFGFR1) or FGFR2 (siFGFR2), as described in Figure S8. (A), FGF9 was added at a concentration of 10ng/ml medium; 20min later cells were lysed and proteins were isolated; 30ng of protein were separated by gel electrophoresis and analysed by western blot; ß-actin was used as housekeeping gene. Intensity of the bands was quantified by using Image J software for densitometry. (B), 48hrs after transfection cells were trypsinized and seeded at a density of 100 (Hep3B) or 250 (HCC3) cells per cm²; 24hrs later cells were treated with 10ng of FGF9 per ml medium. After a period of 10 days cells were fixed, stained and the number and size of clones were evaluated by image analysing software (LUCIA). Number, area and size of clones are illustrated. Data are means ± SEM of 2-3 independent experiments. Statistics by One-Sample t-test: a, p<0.05.*

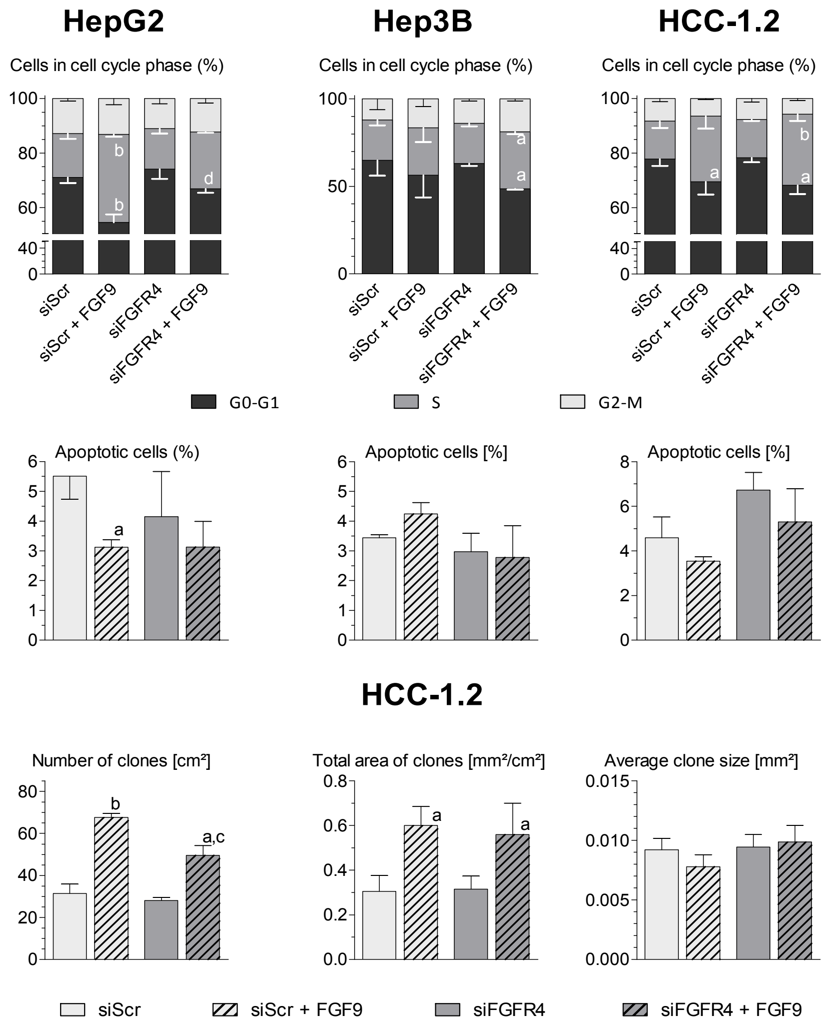

Figure S12. Impact of FGFR4 down-modulation on FGF9-mediated effects in hepatoma/hepatocarcinoma cells. *HepG2, Hep3B and HCC-1.2 cells were transfected either with siSCR or siFGFR4, as described in Figure S8. Cells were treated with 10ng of FGF9/ml medium and 48hrs later FACS determined the percentage of cells in the cell cycle phases or undergoing apoptosis. Further experimental details are given in Methods. To test for clonogenicity, 48hrs after transfection HCC-1.2 cells were trypsinized and seeded at a density of 170/cm^2; 24hrs later cells were treated with FGF9 at 10 ng/ml medium. After a period of 10 days cells were fixed, stained and the number and size of clones were evaluated by image analysing software (LUCIA). Data are means ± SEM of 3 independent experiments. Statistics by unpaired t-test: siSCR vs siSCR+FGF9 or siFGFR4 vs. siFGFR4+FGF9: a, $p < 0.05$; b, $p < 0.01$; siScr +FGF9 vs siFGFR4+FGF9: c, $p < 0.05$; d, $p < 0.01$.*

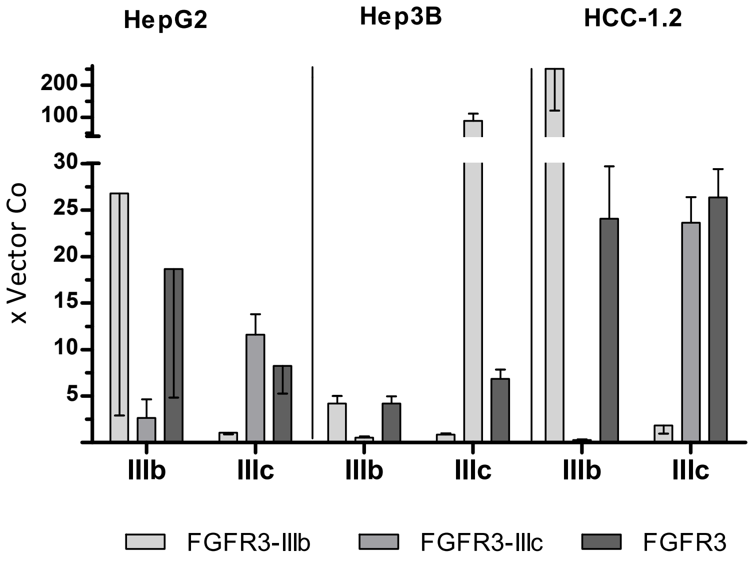

Figure S13. Cell lines with stable overexpression of FGFR3-IIIb or FGFR3-IIIc. *Cell lines with stable overexpression of either FGFR3-IIIb or FGFR3-IIIc were generated by lentiviral transfection. For further details see Paur et al.[2] Expression levels of FGFR3-IIIb, FGFR3-IIIC and total FGFR3 were determined by RT-qPCR. Means ± SD of fold vector control of ≥ 2 independent experiments are given.*

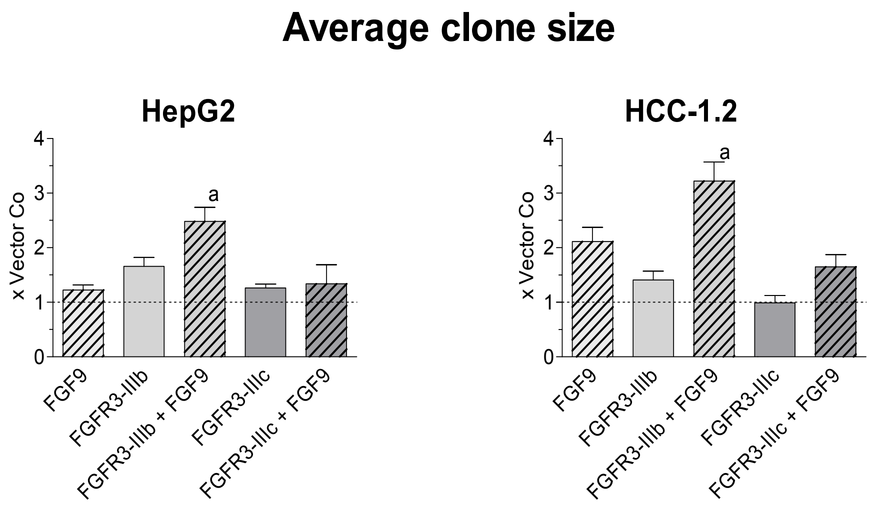

Figure S14. FGF9 enhances clonal growth in hepatoma/hepatocarcinoma cells overexpressing FGFR3-IIIb. *Cells over-expressing the lentiviral control vector (vector Co), FGFR3-IIIb or FGFR3-IIIc had been generated previously.* [2] *For further details see Figure S13. Cells were seeded at densities of 170 cells/cm^2 and 4hrs later were treated with FGF9 at 10ng/ml medium. When clones appeared in controls, cells were fixed, stained and the average clone size was quantified by 'LUCIA G image analyser' (Nikon, Vienna, Austria). Data are expressed as fold vector control and are means ± SEM of 3 independent experiments. Statistics by One-Sample t-test: without vs. with FGF9 treatment: a, $p< 0.05$.*

Table S6. Comparison of two siRNAs silencing FGFR3. *Cells were transfected with either siSCR, siFGFR3 (4392421/s5167, ThermoFisher Scientific) or another siFGFR3 (AM16708, ID 1107280, ThermoFisher Scientific), as described (Figure S8). Cells were treated with 10ng FGF9/ml medium. RT-qPCR, FACS as well as tests for clonogenicity were performed as described in details in Methods. Data are expressed as mean ± SD of ≥2 independent experiments or are single determinations.*

Endpoint	Line/Treatment	siFGFR3 (#4392421/ s5167)	siFGFR3 (AM16708/ ID 1107280)
**FGFR3 Expression (RT-qPCR)**			
FGFR3 mRNA (x siSCR)	HepG2/siFGFR3	0.41 ± 0.22	0.54
	Hep3B/siFGFR3	0.24 ± 0.06	0.31 ± 0.10
	HCC-1.2/siFGFR3	0.17 ± 0.02	0.33 ± 0.43
**Cell Cycle (FACS)**			
% of cells in Go/G1-phase (x siSCR)	Hep3B/siFGFR3	0.90 ± 0.02	1.01 ± 0.02
	Hep3B/FGF9	0.77 ± 0.07	0.60 ± 0.10
	Hep3B/siFGFR3+FGF9	0.93 ± 0.13	0.96 ± 0.05
	HCC-1.2/siFGFR3	1.04 ± 0.05	1.09 ± 0.08
	HCC-1.2/FGF9	0.89 ± 0.09	n.d.
	HCC-1.2/siFGR3+FGF9	0.99 ± 0.03	n.d.
% of cells in S-phase (x siSCR)	Hep3B/siFGFR3	0.96 ± 0.04	1.14 ± 0.28
	Hep3B/FGF9	1.44 ± 0.22	1.93 ± 0.34
	Hep3B/siFGFR3+FGF9	1.17 ± 0.03	1.27 ± 0.10
	HCC-1.2/siFGFR3	0.93 ± 0.21	0.99 ± 0.20
	HCC-1.2/FGF9	1.81 ± 0.78	n.d.
	HCC-1.2/siFGR3+FGF9	1.21 ± 0.08	n.d.
% of cells in G2/M-phase (x siSCR)	Hep3B/siFGFR3	1.17 ± 0.02	1.01 ± 0.03
	Hep3B/FGF9	1.16 ± 0.18	1.01 ± 0.04
	Hep3B/siFGFR3+FGF9	1.04 ± 0.47	0.99 ± 0.01
	HCC-1.2/siFGFR3	0.81 ± 0.21	0.69 ± 0.28
	HCC-1.2/FGF9	0.80 ± 0.12	n.d.
	HCC-1.2/siFGR3+FGF9	0.79 ± 0.17	n.d.
**Apoptosis (FACS)**			
% of apoptotic bodies (x siSCR)	Hep3B/siFGFR3	0.89 ± 0.04	1.04 ± 0.26
	HCC-1.2/siFGFR3	1.34 ± 0.33	1.28 ± 0.1
	HCC-1.2/FGF9	0.85 ± 0.21	n.d.
	HCC-1.2/siFGR3+FGF9	1.21 ± 0.36	n.d.
**Clonogenicity**			
Number of clones (x siSCR)	Hep3B/siFGFR3	0.61 ± 0.07	0.33 ± 0.33
	Hep3B/FGF9	1.51 ± 0.29	n.d.
	Hep3B/siFGFR3+FGF9	0.65 ± 0.23	n.d.
	HCC-1.2/siFGFR3	0.74 ± 0.20	0.50 ± 0.15
	HCC-1.2/FGF9	1.98 ± 0.10	1.53 ± 0.33
	HCC-1.2/siFGR3+FGF9	0.93 ± 0.29	1.12 ± 0.41
Area of clones (x siSCR)	Hep3B/siFGFR3	0.97 ± 0.45	0.93 ± 0.31
	Hep3B/FGF9	1.93 ± 1.00	n.d.
	Hep3B/siFGFR3+FGF9	1.25 ± 0.70	n.d.
	HCC-1.2/siFGFR3	0.93 ± 0.14	1.01 ± 0.20
	HCC-1.2/FGF9	1.68 ± 1.02	1.44 ± 0.21
	HCC-1.2/siFGR3+FGF9	1.08 ± 0.23	0.98 ± 0.32

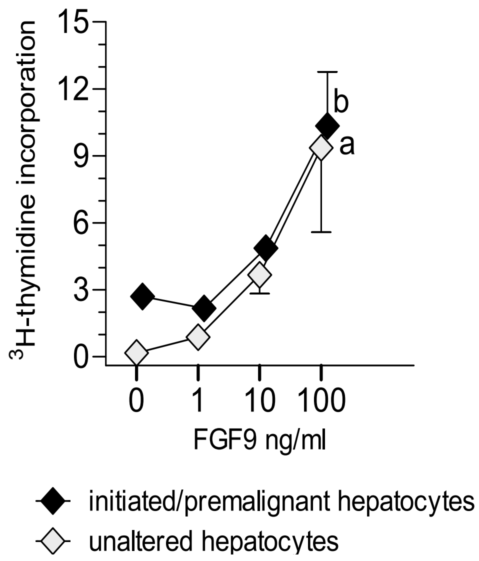

Figure S15. FGF9 stimulates DNA replication of normal and initiated/premalignant rat hepatocytes*. Male Wistar rats received a single application of the genotoxic hepatocarcinogen N-nitrosmorpholine (250mg/kg bw); 21 days later initiated/premalignant hepatocytes (identified by their selective expression of placentar glutathione-S-transferase, GSTp) had developed. Then, livers were perfused with collagenase and the cell suspension obtained was used to separate mesenchymal cells from hepatocytes, as described.[3] Unaltered and initiated/premalignant hepatoctyes were seeded; 4hrs later FGF9 treatment started, was renewed with a medium change after 48hrs and lasted for 72hrs. ^{3}H-thymidine was added 24hrs before harvesting of the cells. Autoradiography served to determine the percentage of nuclei with incorporated ^{3}H-thymidine. In each of the experiments 2000 nuclei of unaltered and 600 nuclei of initiated/premalignant hepatocytes were evaluated.[3] Data are expressed as means ± SEM of 4 independent experiments. Statistics by Kruskal-Wallis test over dose range: a, $p < 0.05$; b, $p < 0.01$.*

References

1. Edmondson HA, Steiner PE. Primary carcinoma of the liver: a study of 100 cases among 48,900 necropsies. Cancer. 1954;7:462–503.

2. Paur J, Nika L, Maier C, et al. Fibroblast growth factor receptor 3 isoforms: Novel therapeutic targets for hepatocellular carcinoma? Hepatology. 2015;62:1767-1778.

3. Nejabat M, Riegler T, Reitinger T, et al. Mesenchyme-derived factors enhance preneoplastic growth by non-genotoxic carcinogens in rat liver. Arch Toxicol. 2018;92:953-966.

4. Gauglhofer C, Sagmeister S, Schrottmaier W, et al. Up-regulation of the fibroblast growth factor 8 subfamily in human hepatocellular carcinoma for cell survival and neoangiogenesis. Hepatology. 2011;53(3):854-864.

5. Sagmeister S, Eisenbauer M, Pirker C, et al. New cellular tools reveal complex epithelial-mesenchymal interactions in hepatocarcinogenesis. Br J Cancer. 2008;99:151-159.
